# Ultraselective sequestration of Li^+^ and Mg^2+^ from brines via a reusable polyoxoniobate-based ion sponge

**DOI:** 10.1126/sciadv.adz7696

**Published:** 2025-12-05

**Authors:** Linfeng Chen, Chenyang Li, Sahand Adibnia, Sizhuo Yang, Jialu Li, Erika Samolova, Andrew Dopilka, Zhiyuan Huang, Raynald Giovine, Xander B. Fleming, Jinghua Guo, Andrew Z. Haddad, Robert Kostecki, Wei Chen, Chaochao Dun, Jeffrey J. Urban

**Affiliations:** ^1^ The Molecular Foundry, Lawrence Berkeley National Laboratory, Berkeley, CA 94720, USA.; ^2^ Department of Materials Design and Innovation, School of Engineering and Applied Sciences, University at Buffalo, The State University of New York, Buffalo, NY 14260, USA.; ^3^ Energy Storage and Distributed Resources Division, Lawrence Berkeley National Laboratory, Berkeley, CA 94720, USA.; ^4^ College of Chemistry, University of California, Berkeley, CA 94720, USA.; ^5^ Advanced Light Source, Lawrence Berkeley National Laboratory, Berkeley, CA 94720, USA.; ^6^ Institute of Physics of the Czech Academy of Sciences, Na Slovance 2, CZ-182 21 Praha, Czech Republic.; ^7^ College of Chemistry, Pines Magnetic Resonance Center – Core Facility, University of California, Berkeley, CA 94720, USA.

## Abstract

Lithium (Li) and magnesium (Mg) are designated as critical mineral materials (CMM) due to their essential roles in clean energy technologies. However, extracting high-purity Li^+^ from brine remains a formidable challenge owing to the presence of Mg^2+^, a physicochemical similar ion that often exists in excess. Here, we introduce a polyoxoniobate-based “Mg-PONb sponge” that enables ultraselective and rapid Li^+^/Mg^2+^ separation across an exceptionally broad range of Mg/Li ratios (0.02 to 200.63). This framework achieves >99.9% Mg^2+^ removal with negligible Li^+^ loss in under 1 min, yielding Li^+^/Mg^2+^ selectivity values exceeding 5000. The sponge demonstrates excellent recyclability, maintaining >99% Mg^2+^ rejection and Li^+^ permeability across five regeneration cycles without structural degradation. Mechanistic investigations reveal that selective Mg^2+^ capture originates from strong coordination with terminal oxygens on the PONb cluster, driving rapid formation of porous Mg-PONb frameworks. This work presents a generalizable, scalable strategy for Li^+^/Mg^2+^ separation and offers a sustainable path toward enhanced Li and Mg recovery from complex brine sources.

## Teaser

A unique Mg-PONb sponge forms on contact enabling rapid and selective Li^+^/Mg^2+^ separation with robust reusability and scalability.

## INTRODUCTION

The rising global demand for lithium (Li) and magnesium (Mg) stems from their essential roles in clean energy technologies, especially battery production (*1*–*3*) and electric vehicle industries (*4*–*6*). Li prices increased nearly 10-fold from 2020 to 2022 (*7*) and demand is projected to rise sevenfold by 2030 (*8*, *9*). While brines account for ~60% of identified Li reserves (*10*, *11*), their exploitation is limited by inefficient extraction methods like the lime-soda process, which are slow, energy and resource intensive, and present a pollution challenge, while offering only modest (<40%) recovery (*12*, *13*). Li extraction from brines conventionally entails a series of purification steps to eliminate more abundant ions such as Na^+^, and Ca^2+^ before isolating the relatively diluted Li^+^ (*14*), while Mg^2+^ is typically removed at a later stage following solar evaporation. Na^+^ is commonly removed via selective evaporation or phase separation (*15*), while Ca^2+^ is typically precipitated as CaCO_3_ (*16*). A core challenge is the selective separation of Li^+^ from Mg^2+^, which has similar hydrated radii and often coexist at high concentrations in natural brines (*13*, *17*–*21*). Conventional methods require repeated precipitation and concentration steps (fig. S1A), causing Li^+^ loss and generating hazardous waste. Although direct lithium extraction technologies, including adsorption, electrochemical, and membrane processes, have emerged (*11*, *13*, *14*, *20*, *21*), they offer potential improvements but are often constrained by their dependency on relatively low Mg-to-Li ratios (MLRs) and often operate at low flux under highly controlled conditions, limiting scalability and broad applicability across diverse brine compositions. Meanwhile, Mg^2+^ itself is a critical material for automotive and aerospace industries, making its co-recovery desirable (*22*).

**Fig. 1.**
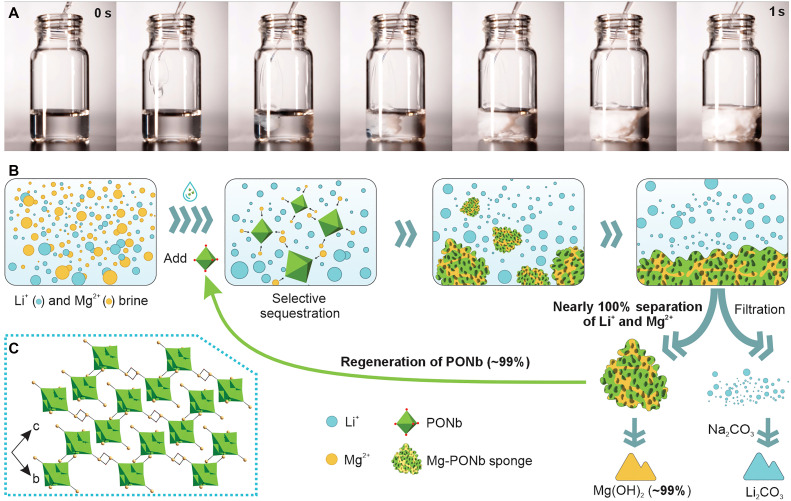
Rapid formation and structural features of the Mg–PONb sponge for selective Mg^2+^ sequestration. (**A**) Time-lapse photographs showing the rapid self-assembly process of the Mg-PONb sponge in brine solutions (left to right). Upon the addition of native-PONb (0.1 M, 1 ml) to an aqueous simulated brine containing MgCl_2_/LiCl (0.1/0.02 M, 5 ml), the solution transitions from transparent to the formation of visible sponge-like aggregates, indicating the successful sequestration of Mg^2+^ ions. This process occurs within 1 second and can be visually monitored, demonstrating the efficient and fast separation of Mg^2+^ from Li^+^ in brine (movie S1). (**B**) Schematic of the PONb-based selective sequestration process. Upon addition of PONb, Mg^2+^ ions are selectively captured from brine, forming a regenerable Mg-PONb sponge. This rapid and selective sequestration process leads to the formation of a visible solid, enabling nearly complete separation of Mg^2+^ from Li^+^. The Mg–PONb sponge can then be isolated by simple filtration and further treated to recover Mg(OH)_2_ with ~99% yield. The PONb material demonstrates excellent reusability, with ~99% regeneration efficiency over repeated cycles. (**C**) The crystalline framework of the resulting Mg-PONb sponge material. Water omitted for clarity. Color code: Nb polyhedral (green), O atoms (red), and Mg atoms (yellow).

Polyoxometalates (POMs) (*23*–*27*), particularly Nb-based POMs (PONb), offer a tunable platform for selective ion separation due to their high surface charge density and structural versatility (*28*–*31*). In this study, we introduce the “Mg-PONb sponge,” a [Nb_6_O_19_]^8−^-based system that selectively sequesters Mg^2+^ from Li^+^, Na^+^, and K^+^ in brines, achieving rapid, near-complete Mg^2+^ recovery and Li^+^ selectivity across a broad MLR conditions (0.02 to 200.63). This approach also exhibits excellent regenerability: over 98.92% of the PONb material can be consistently regenerated, while more than 98.86% of the captured Mg^2+^ is recovered as crystalline Mg(OH)_2_. This previously unknown material and process not only substantially enhance Li^+^/Mg^2+^ separation efficiency but also enable the value-added recovery of Mg^2+^, which is typically discarded as waste (Fig. 1, A and B, and fig. S1B).

## RESULTS

### Mg^2+^-Induced formation of the structurally distinct Mg-PONb sponge

We first prepared a Lindqvist PONb through a one-step hydrothermal approach. Single-crystal x-ray diffraction (SC-XRD) revealed the structure of K_8_{Nb_6_O_19_}•9H_2_O (denoted here as native-PONb), which exhibits a monoclinic *P*2_1_/*c* unit cell containing {Nb_6_O_19_} coordinating with eight K^+^ (figs. S2 to S4 and tables S1 and S2). While our structure adopts a well-defined monoclinic *P*2_1_/*c* unit cell, previous studies have shown that K-PONb materials can exhibit high structural flexibility in the arrangement of K^+^ ions and lattice water around the PONb core (*32*). Upon adding native-PONb to a MgCl_2_ solution, an immediate precipitation of the white powder was observed. Conversely, no changes were observed with the addition of native-PONb to the LiCl solution. Remarkably, even when the MgCl_2_ concentration was diluted to a thousand times lower than that of the LiCl, precipitation still occurred instantly; further investigation by diluting MgCl_2_ and native-PONb by a factor of 10,000 and mixing thoroughly resulted in the successful formation of single crystals after several days (figs. S5 and S6). SC-XRD analysis confirmed the structure of the compound as Mg_4_{Nb_6_O_19_}•16H_2_O (denoted as Mg-PONb), which crystallized in a triclinic *P*$\bar{1}$ unit cell with {Nb_6_O_19_} cluster coordinated by four Mg^2+^ ions (Fig. 1C, figs. S6 to S8, and tables S1 and S3). In this structure, four crystallographically distinct Mg^2+^ sites are observed. Among them, Mg1 and Mg2 are fully occupied, while Mg3 and Mg4 exhibit 0.5 occupancy and are modeled using PART 1. The complementary positions are occupied by disordered water molecules represented by PART 2. Mg2 and Mg4 serve as bridging ions between adjacent {Nb_6_O_19_} clusters, whereas Mg1 and Mg3 are terminally bound and decorate the cluster peripheries without contributing to intercluster linkages. Because of the partial occupancy and positional disorder of Mg3 and Mg4, the overall structure exhibits a partially connected one-dimensional framework that is best described as a mixed configuration of chain-like and dimeric motifs. As controls, we also analyzed single-crystal Li-PONb (HLi_7_{Nb_6_O_19_}•13H_2_O) and Na-PONb (H_2_Na_6_{Nb_6_O_19_}•15H_2_O) crystals. The structure of Li-PONb crystallized in an *R*$\bar{3}$ unit cell (figs. S6, S9, and S10 and tables S1 and S4) and Na-PONb crystallized in an orthorhombic *Pnnm* unit cell (figs. S6 and S11 to S13 and tables S1 and S5). In Li-PONb, four crystallographically distinct Li^+^ sites were identified. Li1 and Li4 are disordered and mutually exclusive (each modeled at 0.5 occupancy), corresponding to two alternative structural motifs. Li1 and O6 participate in the formation of a water-coordinated, adamantane-like cluster, which is further connected to a chain composed of Li2 and Li3. Alternatively, Li4 and O5 form a more extended chain of octahedrally coordinated Li ions. In both disorder models, Li^+^ ions are solvated and bridged by water molecules, with no direct bonding to the {Nb_6_O_19_}^8−^ cluster. This structural arrangement is consistent with previously reported lithium hexaniobate frameworks (*33*, *34*), and the current disorder model provides a good fit to the crystallographic data. For Na-PONb, this Na-PONb structure represents only the third reported single-crystal Na-containing hexaniobate. Compared to previously reported Na analogs, it contains fewer Na^+^ ions per {Nb_6_O_19_}^8−^ unit and was obtained via postsynthetic ion exchange from native-PONb. Despite these differences, the Na^+^ coordination geometry and linkage pattern are consistent with prior models (*35*, *36*).

**Fig. 2.**
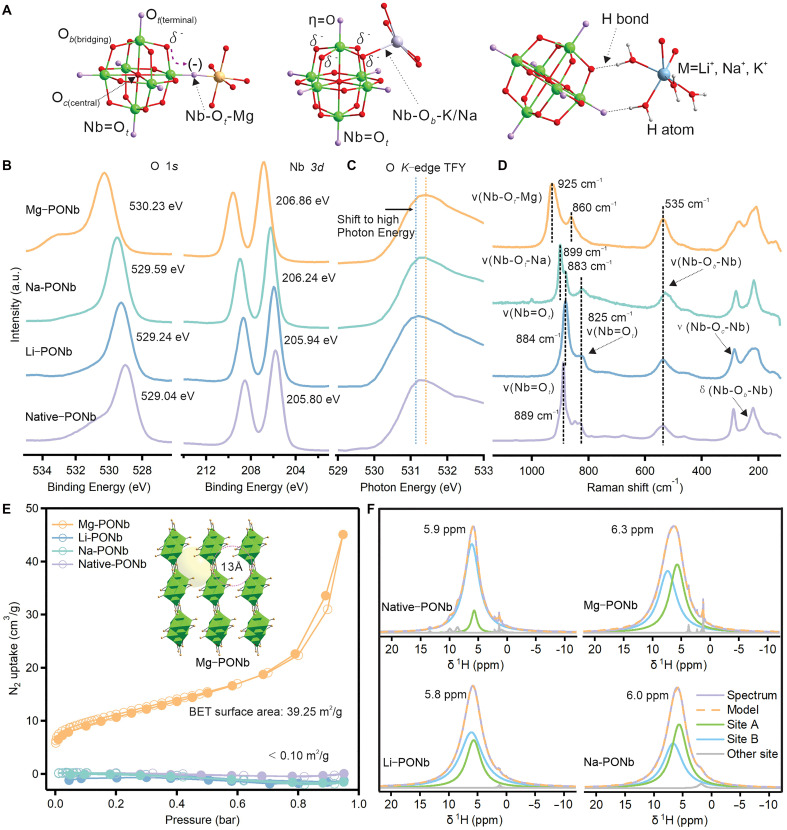
Structural, electronic, and porosity features of M-PONb materials. (**A**) Ball-and-stick representation of different bond connections with PONb: Nb─O*_t_*─M (left), Nb─O*_b_*─M (middle), and Nb─O─H bond (right). Water omitted for clarity. Color code: Nb (green), terminal O atoms (pink), rest of O atoms (red), Mg (orange), K (purple), and Li (blue). (**B**) O 1*s* (left) and Nb 3*d* (right) XPS of M-PONb. (**C**) O K-edge TFY of M-PONb. (**D**) Raman spectra of M-PONb. (**E**) N_2_ adsorption isotherm plot of M-PONb. (**F**) ^1^H solid-state NMR spectra and the corresponding devolution of ^1^H line shapes of M-PONb. (M = K^+^, Li^+^, Na^+^, and Mg^2+^). Here, site A is associated with interactions between H_2_O and the POM cluster, while site B is associated with interactions between H_2_O and M. The observed ^1^H chemical shift of water in M-PONb is indicated for each spectrum. More details about the signal deconvolution can be found in note S1.

Through the analysis of Mg-PONb, native-PONb, Na-PONb, and Li-PONb, three distinct types of bond connections were identified (Fig. 2A). The first type involves direct bonding to terminal oxygen (O*_t_*), the second type involves bonding to bridging oxygen (O*_b_*), and the third type involves metal-cluster connection via hydrogen bonding (H-bond). The bonding modes of these four different crystals and their corresponding calculated binding energies were summarized (table S6). In Li-PONb, the metals coordinate with the cluster exclusively through H-bonds. In native-PONb, the metals primarily bond with O*_b_*. Na-PONb exhibits an even distribution of the three connection types, while in Mg-PONb, the metals connect solely to O*_t_*.

X-ray photoelectron spectroscopy (XPS) was conducted to further analyze the bond coordination. The O 1*s* and Nb 3*d* spectra of native-PONb, Li-PONb, Na-PONb, and Mg-PONb are shown in Fig. 2B. The binding energies attributed to Nb-O were measured at 529.0, 529.2, 529.5, and 530.2 eV, respectively. A similar peak shift order was observed in the Nb 3*d* spectra. Native-PONb and Li-PONb retained their peaks at 205.80 and 205.94 eV, respectively, while Na-PONb and Mg-PONb shifted to 206.24 and 206.86 eV, respectively. This behavior is primarily attributed to the coordination environment of O*_t_* in the different PONb structures. In Li-PONb and native-PONb, Li^+^ and K^+^ ions exhibit minimal coordination with O*_t_*, allowing the Nb═O*_t_* bonds to remain predominantly double bonds. This results in a relatively uniform electron cloud distribution over the bridging O atoms (Fig. 2A). However, in Na-PONb and Mg-PONb, the metal ions coordinate with O*_t_*, causing the negative charge to be distributed across five single Nb─O bonds. The bond to the central O site is clearly elongated and weak, effectively rendering it negligible and resulting in the anion site being localized on the O*_t_* coordinated to the metal (*37*). Consequently, the overall O 1*s* peak shifts to higher binding energies. A similar shift is observed in the Nb 3*d* spectra due to analogous coordination. These observations are consistent with the phenomena observed in the SC-XRD analysis and bond classification in table S6. In addition, O K-edge x-ray absorption spectra (XAS) was also measured (Fig. 2C). A similar shift, with Mg-PONb showing a higher photon energy compared to native-PONb and Li-PONb, was also observed in the O K-edge total fluorescence yield (TFY). This further confirms the presence of the unique and strong Nb─O*_t_*─Mg bonds in Mg-PONb.

Raman spectroscopy can distinguish the vibrational bands of different oxygen atoms within PONb, providing a deeper understanding of their bond coordination (*38*–*40*). Figure 2D reveals the consistent highly symmetric octahedral {Nb_6_O_19_} cluster configuration, as indicated by similar vibrational bands for Nb-O*_c_*-Nb and Nb-O*_b_*-Nb in the 200 to 600 cm^−**1**^ regions (*38*). The sharp peaks in the >800 cm^−**1**^ region were assigned to vibrational bands of Nb-O*_t_*, exhibiting notable differences among the four samples (*39*). Intense peaks at 889 cm^−**1**^ in both native-PONb and Li-PONb are attributed to the Nb═O*_t_* bond vibrational modes. For Na-PONb, two distinct peaks were observed around 900 cm^−**1**^: one at 899 cm^−**1**^, associated with the Nb-O*_t_*-Na vibrational mode, and another at 883 cm^−**1**^, corresponding to the Nb═O*_t_* vibrational mode (*38*, *39*). In addition to these high-frequency peaks, a prominent band near 825 cm^−**1**^ is consistently observed in the spectra of native-, Li-, and Na-PONb. This peak is assigned to the symmetric stretching of Nb═O*_t_* bonds within the PONb. These observations are consistent with bond connections elucidated through crystal structure analysis (figs. S2, S7, S9, and S11). Crystallographic data indicate the presence of two Nb─O*_t_*─Na bond in Na-PONb (2.45 to 2.47 Å, table S5), which contrasts with the native-PONb structure, featuring only a K─O*_t_* bond over 3.24 Å (fig. S2 and table S2), and Li-PONb, which lacks a Li─O*_t_* bond (fig. S9 and table S4). The substantial spectral shifts were observed in Mg-PONb, with the intense peaks at 925 and 860 cm^−**1**^, attributed to the Nb-O*_t_*-Mg vibrational mode. This also aligns with crystallographic data (fig. S7 and table S3), which confirm that all Mg^**2**+^ ions are coordinated to O*_t_* and exhibit four robust Nb─O*_t_*─Mg bonds, each approximately 2.0 Å in length. The shift to higher frequencies indicates stronger interactions between cations and {Nb_6_O_19_} cluster, with the Nb-O*_t_* Raman shift directly correlated to the M─O*_t_* bond distance (table S6). Such frequency shifts are also evident in the {Nb_10_} cluster (*40*). Another key difference is the broadening of Mg-PONb Raman peaks, likely indicating poor crystallinity, which is also consistent with the PXRD results for Mg-PONb (fig. S6) (*41*). This is inferred from the immediate precipitation, which prevents large crystal growth and results in ultrafine crystals.

We posit that the unique open network structure of Mg-PONb is driven by Mg-bonding at O*_t_* sites which leads to formation of Mg-PONb sponge; these condensed structures lead to the immediate precipitation of ultrafine Mg-PONb crystals. Consistent with formation of these larger, mesoscopic open network structures, Mg-PONb exhibits substantially enhanced (~400-fold increase) porosity compared to native-PONb, Li-PONb, and Na-PONb, which retain their cluster identity even after metal binding. The Brunauer-Emmett-Teller (BET) surface area was approximately less than 0.1 m^2^/g for Na-PONb, Li-PONb, and native-PONb, confirming that these samples are almost nonporous (Fig. 2E). However, after the introduction of Mg^2+^, the BET surface area markedly increased to 39.25 m^2^/g. This substantial increase corresponds with the porous sponge structure observed through SC-XRD analysis and small crystals size from PXRD and Raman.

We further use quantitative ^1^H spin-echo solid-state nuclear magnetic resonance (ssNMR) measurements under fast magic angle spinning conditions to reveal detailed insights into the chemical structure and water molecule distribution within PONbs, especially the unique porosity of Mg-PONb. As can be seen in Fig. 2F and fig. S14, all four PONbs variants exhibited a primary water resonance at approximately 6 parts per million (ppm) (*32*, *42*), indicative of a highly charged niobate cluster environment, similar to Brønsted acid sites in zeolites (*43*). Mg-PONb showed a distinct shift in water resonance to 6.3 ppm, slightly higher than the others, suggesting a unique interaction within its structure. Furthermore, spectral deconvolution confirmed the presence of two distinct proton environments related to water molecules in these PONbs. The first site, A, maintains its chemical shift across all four PONbs at 5.7 ppm and was assigned to H_2_O-PONb cluster while the second site, B, was downfield to site A and found in a range of chemical shifts across PONbs (from 6.1 to 7.4 ppm). These variations in chemical shift demonstrate different bonding interactions between water and cations (Li^+^, Na^+^, K^+^, and Mg^2+^). Table S7 reveals that native-PONb has a disproportionate distribution of water sites, with a ratio of 10.8:1 in favor of site B, while the other three PONbs (Mg-PONb, Li-, and Na-) exhibit a more balanced distribution near 1:1. This observation agrees with the crystal structures obtained from SC-XRD (figs. S2 to S4), where native-PONb has eight K^+^ centers surrounding PONb that limit interactions between water molecules and the cluster. The limited presence of site A protons in native-PONb also aligns with its acidic O*_b_*-H proton resonances, indicating fewer available PONb sites for water interaction due to occupied hydroxyl groups. In contrast, Mg-PONb shows the most substantial increase in site A content, 17-fold, reflecting the reduction in acidic proton resonances. Overall, Mg-PONb displayed the most substantial variation, with a marked increase in one type of proton environment, correlating with its increased porosity and water content, up to three times that of native-PONb. This enhanced water content in Mg-PONb is attributed to its large surface area and pore size, which facilitates more extensive water molecule adsorption, as supported by BET and SC-XRD analyses. More details on the NMR analysis are provided in note S1.

### Ultraselective Li^+^/Mg^2+^ separation over a broad MLR window

**Fig. 3.**
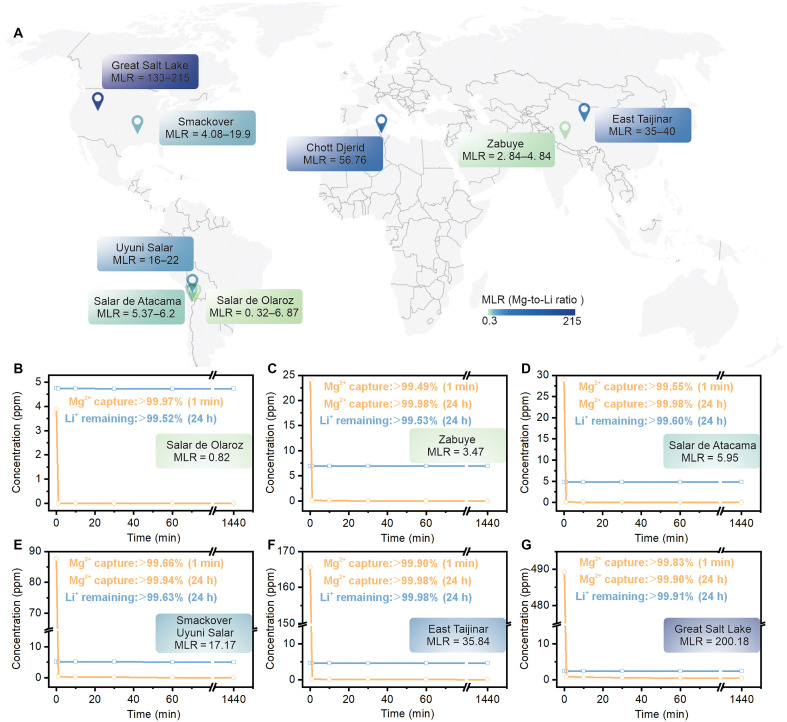
Global relevance and validation of Mg–PONb performance across diverse brine compositions. (**A**) Word map illustrating the Mg-to-Li ratio (MLR) in brines globally. (**B** to **G**) Results from ICP-OES analysis of synthetic Li/Mg brine, designed to replicate the MLR of major brine sources worldwide.

Because of its unique structural features such as strong Nb─O*_t_*─Mg bonds and substantially enhanced surface area, Mg-PONb rapidly precipitates, showing great promise for efficient Li and Mg separation. The performance of native-PONb materials in the selective separation of Li^+^/Mg^2+^, Na^+^/Mg^2+^, and K^+^/Mg^2+^ was evaluated using inductively coupled plasma optical emission spectrometry (ICP-OES). Given the substantial global variability in MLR of brines (Fig. 3A) and the considerable concentration differences even among brines with identical MLRs (*18*), 22 laboratory-prepared simulated binary salt mixture composed of MgCl_2_ and LiCl were developed to mimic the compositions of major salt lakes (tables S8 to S10).

First, 11 different MLRs ranging from 0.02 to 200 of binary Li^+^/Mg^**2**+^ salt mixtures with a total salt concentration of less than 1000 ppm were tested (Fig. 3, B to G, fig. S17, and table S8). The obtained mixtures at different time intervals were compared with the initial simulated salt mixtures. The concentration of Li^+^ remained in the supernatant almost constant within the 24-hour testing period, whereas the concentration of Mg^**2**+^ in the supernatant markedly decreased within a short period, with almost no Mg^**2**+^ detectable after 24 hours. Specifically, for these 11 different MLR simulated feed mixtures with relatively low concentrations, an average of 99.74% of Li^+^ remained in mixtures after 24 hours, while an average of 0.07% of Mg^**2**+^ was detectable. The average Li^+^/Mg^**2**+^ selectivity for these 11 reactions reached 3243, with the highest exceeding 5000, marking the most exceptional Li^+^/Mg^**2**+^ selectivity reported to date (fig. S20). This material also represents the first to maintain such high selectivity across a wide range of MLRs (varying by over 10000-fold).

To further investigate the potential for Li^+^/Mg^**2**+^ separation under higher feed concentrations, closer to actual industrial conditions, total salt concentrations of ~1000 to 3000 ppm and 7000 to 30,000 ppm were also tested. For higher feed concentrations, five different mid-range concentration simulated feed mixtures (MLR = 0.27 to 200.63; fig. S18 and table S9) and six different high concentrations simulated feed mixtures (MLR = 0.036 to 199.85; fig. S19 and table S10) were prepared. Similar to the low concentration scenario, in the mid-range concentration (~1000 to 3000 ppm), over 99.69% of Li^+^ in the supernatant remained in mixtures after 24 hours, while less than 0.36% of Mg^**2**+^ was detectable, achieving a selectivity of 1523. At high concentrations (7000 to 30,000 ppm), even with the addition of higher concentrations of native-PONb material, less than 0.65% of Li^+^ was lost, and over 99.91% of Mg^**2**+^ was completely excluded. The average Li^+^/Mg^**2**+^ selectivity in this scenario also exceeded 1709. These ICP-OES results robustly confirm the superior Mg^**2**+^ extraction capability of native-PONb materials and their high selectivity for Li^+^/Mg^**2**+^, underscoring their potential for efficient Li^+^/Mg^**2**+^ separation in diverse brine compositions.

Native-PONb exhibits the highest reported Li^+^/Mg^**2**+^ selectivity across an exceptionally broad MLR range, along with high Li^+^ purity and minimal Li^+^ loss, outperforming all benchmarked separation technologies (fig. S20 and tables S11). Native-PONb also demonstrates near-complete Mg^**2**+^ removal from Na^+^- and K^+^-containing mixtures with minimal co-capture, confirming its robust applicability for alkali-Mg^**2**+^ separations (figs. S21 and S22 and tables S12 and S13).

### Mechanistic insights and Long-term Regenerability

**Fig. 4.**
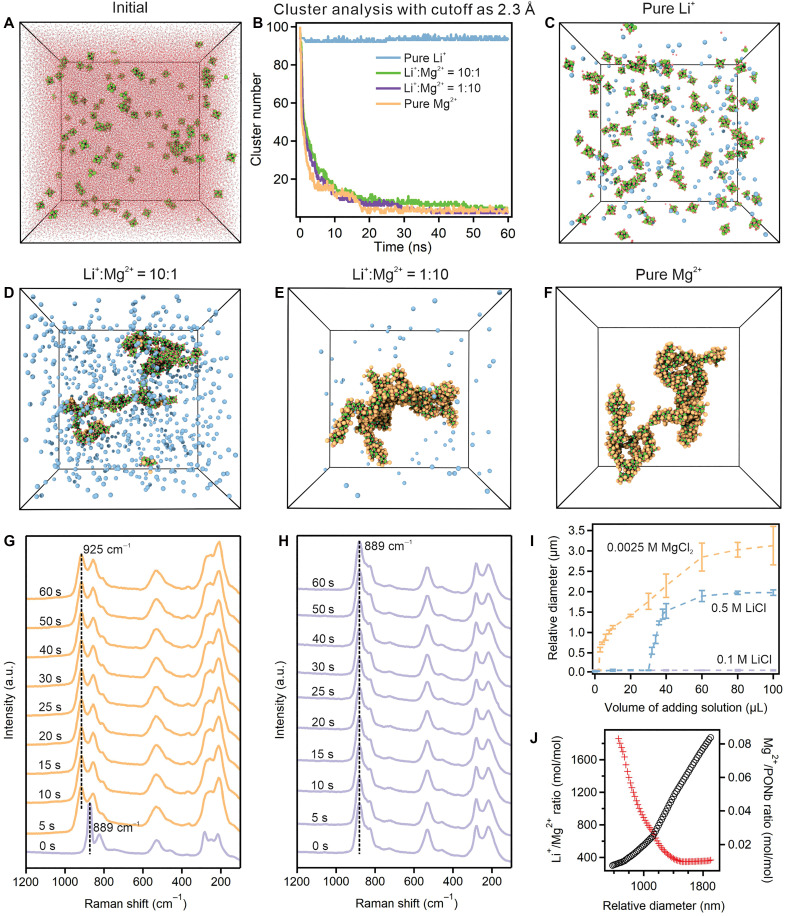
Ion-specific aggregation and interaction behavior of PONb with Li^+^ and Mg^2+^ revealed by simulations and experiments. (**A**) Representative simulation snapshots depicting the initial configuration of native-PONb molecules in aqueous mixtures prior to the addition of various metal cations. (**B**) Aggregate state analysis based on cluster formation, using a cutoff distance of 2.30 Å. Representative snapshots illustrate the system following the addition of (**C**) pure Li^+^ ions, (**D**) a mixture of Li^+^ and Mg^2+^ ions at a 10:1 ratio, (**E**) a mixture of Li^+^ and Mg^2+^ ions at a 1:10 ratio, and (**F**) pure Mg^2+^ ions. Color code: Nb (green), O (red), Mg (orange), K (purple), and Li (blue). In situ Raman spectroscopy results following the addition of (**G**) Mg^2+^ ions and (**H**) Li^+^ ions within 60 s. (**I**) Dynamic light scattering (DLS) analysis comparing the effects of different amount of Li^+^ and Mg^2+^ ions. (**J**) DLS analysis comparing the effects of different amounts of Li^+^ and Mg^2+^ ions.

To further elucidate the kinetic mechanism of Li-Mg extraction using PONb-based methods (Fig. 4, A to F), we also used molecular dynamics (MD) simulations. The computational results demonstrated a strong preference for PONb clustering in the presence of Mg^**2**+^ ions, leading to almost complete adsorption of Mg^**2**+^ and substantial aggregation, while interactions with Li^+^ ions were weak, resulting in minimal adsorption and no observable aggregation. These observations are consistent with prior MD studies by Segado *et al.* (*44*), which reported that Li^+^ interacts with [Nb_6_O_19_]^8−^ via bridging oxygens without inducing aggregation. The computational results indicate that native-PONb exhibited remarkable selectivity and sensitivity for both Li^+^ and Mg^**2**+^ ions. These findings are consistent with those obtained from dynamic light scattering (DLS), which also indicated that native-PONb showed high selectivity and sensitivity for Li^+^, Na^+^ and Mg^**2**+^ ions: Mg^**2**+^ ions caused substantial aggregation of at least 1000 nm even at very small amount (8 μl, 0.0025 M) once injected, unlike the changes observed until thousands of times of Li^+^ (40 μl, 0.5 M) and Na^+^ (40 μl, 0.5 M) ions, suggesting lower solubility and higher reactivity of native-PONb with Mg^**2**+^ ions (Fig. 4, I to J, and figs. S23 to S25). We also used DLS measurements to investigate the effect of pH on the solubility behavior of Li^+^ and Mg^**2**+^ in native-PONb solutions (fig. S26). The results show that at lower pH values (*7*–*9*), the solubility of both ions increases, but Li^+^ is more strongly affected than Mg^**2**+^, leading to improved Li/Mg selectivity. These findings suggest that native-PONb maintains robust Li^+^/Mg^**2**+^ selectivity across a broad pH range (*7*–*14*), highlighting its potential applicability under various brine conditions.

Reaction time is a critical factor in the efficiency of Li^+^ and Mg^2+^ separation. Traditional methodologies typically require several minutes to hours for completion (*45*–*47*). On the contrary, our study found that over 99.9% Mg^2+^ can be captured within 1 min, with Li^+^ concentrations remaining above 99.0%, even across a wide MLR range of 0.27 to 200.64 (Figs. 1, A to C, and 3, B to G; and figs. S17 to S19). In cases of extremely low MLR (<0.05), Mg^2+^ rejection rates reached approximately 90% within the first hour, escalating to 99.9% after 24 hours. This rapid separation is underscored by in situ Raman spectroscopy, which detected a clear spectral shift from 889 to 925 cm^−1^ within 5 s after adding MgCl_2_, indicating a quick structural transition of native-PONb. The stability of the 889 cm^−1^ peak with the addition of LiCl confirms the absence of interaction with Li^+^ (Fig. 4, G and H). These observations, coupled with video documentation (fig. S28 and movie S2) and MD simulation results (movies S3 to S6), demonstrate that native-PONb enables fast and efficient Li^+^/Mg^2+^ separation, markedly reducing the required reaction time compared to existing methods.

**Fig. 5.**
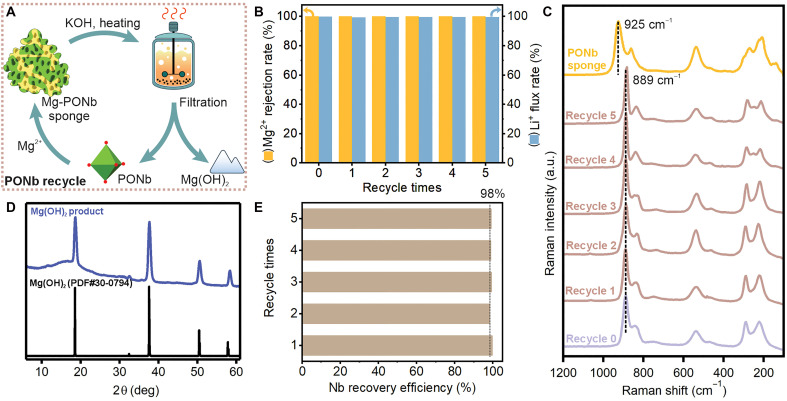
Regeneration and long-term stability of the Mg-PONb sponge system for repeated Li^+^/Mg^2+^ separations. (**A**) Schematic of the regeneration process. After the selective sequestration of Mg^2+^ by PONb to form a Mg-PONb sponge, treatment with KOH and heating releases Mg^2+^ as Mg(OH)_2_, allowing recovery of active PONb. (**B**) Mg^2+^ rejection rate and Li^+^ flux rate over five separation-regeneration cycles, showing stable performance without degradation. A yellow box indicates Mg^2+^ and a blue box indicates Li^+^ to clearly distinguish the two in the plot. (**C**) Raman spectra of the PONb sponge after each cycle. (**D**) XRD pattern of the solid collected during the regeneration step, matching the reference pattern for Mg(OH)_2_ (PDF no. 30-0794), confirming that Mg(OH)_2_ is formed as a by-product during PONb regeneration. (**E**) Nb recovery efficiency across five regeneration cycles, consistently achieving over 98%, highlighting the excellent recyclability of the system. The dotted line represents the 98% reference level.

Last, to evaluate the regeneration capability of PONb after Mg^2+^ sequestration, we conducted a cyclic regeneration-reuse test based on the conversion of Mg-PONb sponge back to its active form (Fig. 5A). After the selective sequestration of Mg^2+^ by PONb, forming a Mg-PONb sponge, the solid was treated with KOH under heating, allowing complete release of Mg^2+^ as crystalline Mg(OH)_2_ with a conversion efficiency around 99%, and enabling recovery of the PONb precursor. The high purity and crystallinity of the resulting Mg(OH)_2_ facilitate its potential reuse in downstream applications. The regenerated PONb was then reused for Li^+^/Mg^2+^ separation. As shown in Fig. 5B and table S14, the regenerated PONb exhibits Mg^2+^ rejection rates and Li^+^ flux values both over 99%, demonstrating separation performance nearly identical to that of the native material across five cycles. Raman spectroscopy further confirmed the structural integrity of the regenerated PONb, with characteristic Nb═O vibrational peaks at 925 and 889 cm^−1^ retained throughout the regeneration cycles (Fig. 5C). XRD analysis of the solid by-product revealed crystalline Mg(OH)_2_ (PDF no. 30-0794) formed during regeneration (Fig. 5D and table S15), while Nb recovery efficiency remained above 98.1% in all cases (Fig. 5E and table S15), validating the high-fidelity regeneration and reusability of the PONb system. This excellent recyclability substantially mitigates concerns regarding the cost of Nb, as the PONb can be efficiently reused with minimal loss, supporting long-term viability for potential large-scale application.

## DISCUSSION

We report the Mg-PONb sponge, a Nb-based POM material that enables rapid and highly selective Li^+^/Mg^2+^ separation across a broad range of brine compositions. Spectroscopic, crystallographic, and computational analyses reveal that strong Mg^2+^ coordination to terminal oxygen sites in the PONb cluster drives the formation of a structurally distinct porous framework, enabling >99.9% Mg^2+^ exclusion within seconds while achieving nearly 100% separation efficiency for both Li^+^ and Mg^2+^. In contrast to conventional evaporation-based methods, the Mg-PONb sponge operates with exceptional speed, selectivity, and scalability. The material maintains consistent structural integrity and separation performance over at least five regeneration cycles, with Nb recovery efficiency remaining around 98.92% and over 98.86% of Mg^2+^ consistently recovered as Mg(OH)_2_. These results demonstrate a robust, selective sequestration mechanism unique to PONb frameworks and establish their practical potential for sustainable ion separations in complex aqueous systems.

## MATERIALS AND METHODS

### Materials

All chemicals were used without further purification. Hydrochloric acid (36.5 wt %, complementary metal-oxide semiconductor grade) was purchased from J.T. Baker. Lithium chloride (99.9%) and methanol (99.90%) were obtained from Fisher Chemical. Magnesium chloride (99.9%), niobium(V) oxide (99.99%), nitric acid (70 wt %, ACS reagent), potassium chloride (99.5%), potassium hydroxide (99.9%), and sodium chloride (>99%) were all purchased from Sigma-Aldrich. Ultrapure water was collected from a MilliQ Advantage A10 system.

### Preparation of native-PONb (K_8_[Nb_6_O_19_]•9H_2_O)

A mixture consisting of Nb_2_O_5_ (0.2 g, 0.75 mmol) and KOH pellets (2.24 g, 40 mmol) was combined with 20.0 ml of deionized (DI) water in a 43-ml Teflon-lined acid digestion vessel. This reaction mixture was stirred for approximately 5 min at room temperature to facilitate dissolution. Subsequently, the vessel was sealed in an autoclave reactor and heated to 200°C for 72 hours. Upon cooling to room temperature, the supernatant was collected, and 60 ml of methanol (MeOH) was added. The mixture was then stirred for 30 min to precipitate out the product. The resultant white precipitate was isolated via filtration and washed three times with 10.0 ml of cold 1:1 MeOH/DI water solution. The purified white crystals were finally collected by filtration, followed by drying for 3 hours.

### Regeneration of native-PONb and recovery of Mg(OH)_2_

A mixture consisting of Mg-PONb (500.11 mg, 0.40 mmol) and KOH pellets (5.60 g, 100 mmol) was combined with 50.0 ml of DI water in a 100-ml Teflon-lined acid digestion vessel. When smaller amounts of Mg-PONb were used, the quantities of KOH and water were proportionally reduced. This reaction mixture was stirred for approximately 5 min at room temperature to facilitate dissolution. Subsequently, the vessel was sealed in an autoclave reactor and heated to 200°C for 24 hours. After naturally cooling to room temperature, the reaction suspension was separated by centrifugation. The resulting solid and supernatant were collected separately. The solid was washed with deionized water and dried under vacuum for 3 hours to yield crystalline Mg(OH)_2_. The supernatant was subsequently treated with 200 ml of MeOH to induce precipitation. The resulting solid was washed three times with 20.0 ml of cold 1:1 MeOH/DI water solution and then dried under vacuum for 3 hours to yield the regenerated PONb as a white powder.

## Supplementary Materials

### The PDF file includes:

Figs. S1 to S29 Tables S1 to S15 Supplementary Text Legends for movies S1 to S6 Legends for data S1 to S4 References
