# Supplementary material for: Ultraselective sequestration of Li+ and Mg2+ from brines via a reusable polyoxoniobate-based ion sponge
Source: Sci Adv. 2025 Dec 5;11(49):eadz7696. doi: 10.1126/sciadv.adz7696 (PMC12680045; doi:10.1126/sciadv.adz7696)
Supplement: Supplementary file 1 — Figs. S1 to S29 Tables S1 to S15 Supplementary Text Legends for movies S1 to S6 Legends for data S1 to S4 References [file sciadv.adz7696_sm.pdf]

Science Advances
AAAS

Supplementary Materials for

Ultraselective sequestration of Li^{+} and Mg^{2+} from brines via a reusable polyoxoniobate-based ion sponge

Linfeng Chen *et al.*

Corresponding author: Chaochao Dun, cdun@lbl.gov; Jeffrey J. Urban, jjurban@lbl.gov

Sci. Adv. **11**, eadz7696 (2025)
DOI: 10.1126/sciadv.adz7696

The PDF file includes:

Figs. S1 to S29
Tables S1 to S15
Supplementary Text
Legend for movies S1 to S6
Legend for data S1 to S4
References

Other Supplementary Material for this manuscript includes the following:

Movies S1 to S6
Data S1 to S4

Supplementary Scheme, Tables and Figures

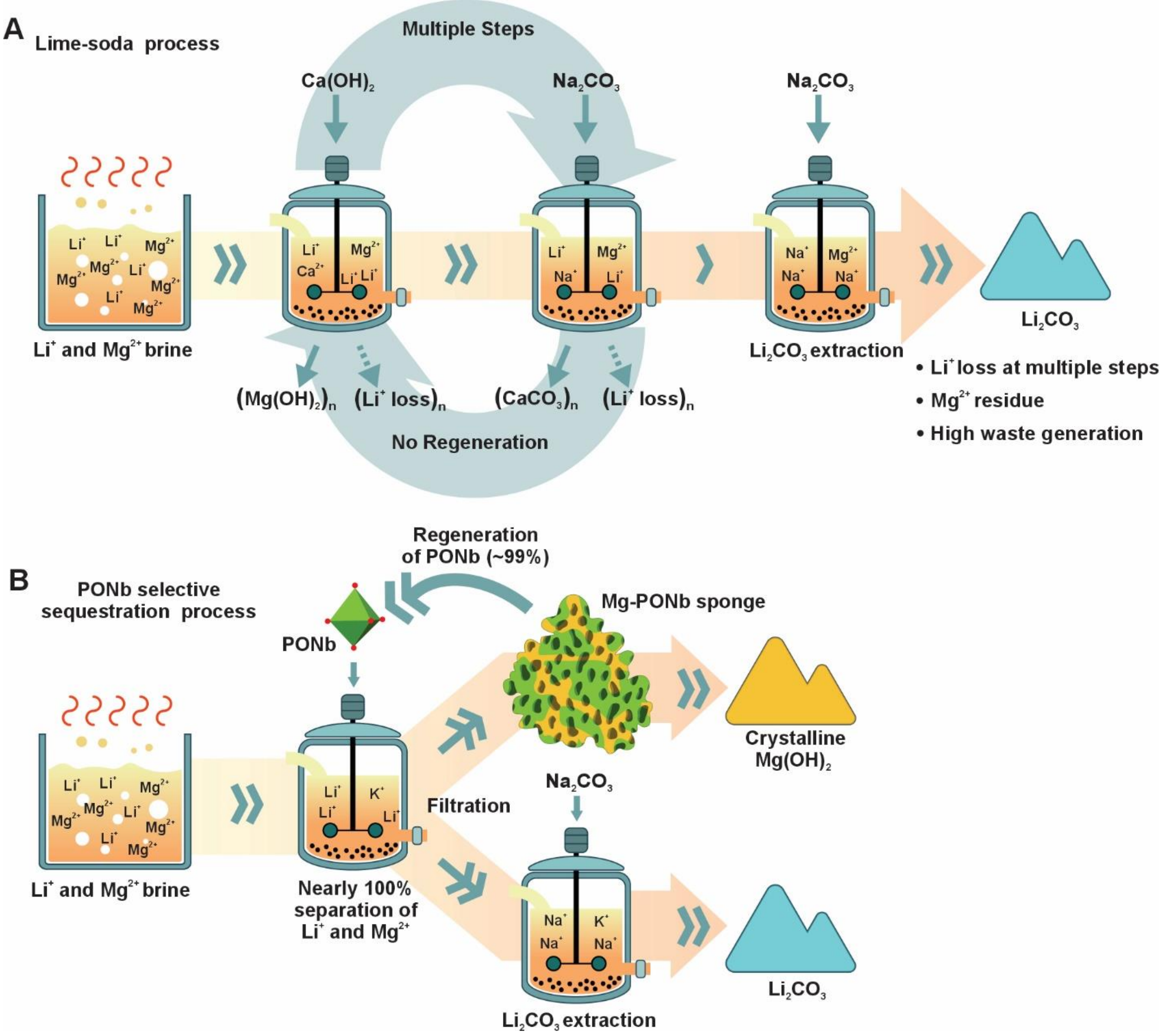

Figure S1. Comparison between the industrial lime-soda process and the PONb-based selective sequestration process. **(A)** Schematic illustration of the conventional lime-soda evaporation method for Li^+ recovery from mixed Li^+/Mg^{2+} brines. The process involves multiple sequential precipitation steps (typically 5–10 cycles), where $Ca(OH)_2$ and Na_2CO_3 are alternately added to remove Mg^{2+} and Ca^{2+} ions via the formation of $(Mg(OH)_2)_n$ and $(CaCO_3)_n$ precipitates. However, this process suffers from significant lithium loss at each stage due to co-precipitation and inefficient separation, lacks a regeneration mechanism, and produces large volumes of solid waste. **(B)** In contrast, the PONb-based selective sequestration process enables rapid and nearly complete separation of Mg^{2+} and Li^+ from brine in a single step. Upon addition of PONb, Mg^{2+} ions are selectively captured to form a Mg–PONb sponge, leaving Li^+ and K^+ in the supernatant. After filtration, Mg^{2+} can be recovered as crystalline $Mg(OH)_2$ (~99% yield), and the Li^+-rich filtrate can be further processed with Na_2CO_3 to yield Li_2CO_3, following literature-reported procedures. Importantly, the PONb material can be regenerated with ~99% efficiency, enabling multiple-use cycles and significantly reducing waste generation.

Table S1. Crystallographic parameters of coordination networks

	**Native-{Nb_6O_{19}}**	**Mg-{Nb_6O_{19}}**	**Li-{Nb_6O_{19}}**	**Na-{Nb_6O_{19}}**
Empirical formula	$H_{13.83}K_{7.97}$ $Nb_6O_{35.44}$	$C_{2.5}Mg_3$ $Nb_6O_{39.5}$	$H_{18}Li_7$ Nb_6O_{46}	$H_{26}Na_6$ Nb_6O_{32}
Formula weight (g/mol)	1450.18	1292.42	1360.2	1233.60
Crystal size (mm^3)	0.2 × 0.15 × 0.1	0.2 × 0.15 × 0.1	0.15 × 0.13 × 0.1	0.15 × 0.12 × 0.1
Crystal system	monoclinic	triclinic	trigonal	Orthorhombic
Space group	$P2_1/c$	$P\bar{1}$	$R\bar{3}$	*Pnnm*
a (Å)	8.9534(14)	11.003(2)	11.9894(9)	12.0538(10)
b (Å)	11.744(2)	11.1882(19)	11.9894(9)	12.6260(10)
c (Å)	17.927(3)	15.878(3)	23.798(2)	10.0226(7)
α (°)	90	100.587(7)	90	90
β (°)	97.973(6)	109.191(8)	90	90
γ (°)	90	90.392(7)	120	90
Volume (Å^3)	1866.8(5)	1809.9(6)	2962.6(5)	1525.2(2)
Z	2	2	3	4
ρ_{calc} (g/cm^3)	2.580	2.372	2.287	2.686
(mm^{-1})	2.776	2.010	1.813	2.377
Goodness-of-fit on F^2	1.374	1.239	1.389	1.085
Reflections collected	79121	88574	34425	68641
Independent reflections	3827	6386	1342	1656
R_1	0.0448	0.0528	0.0359	0.0171
wR_2	0.0927	0.1224	0.1001	0.0463
CSD #	2371924	2371923	2371921	2371922

Radiation: Mo K_α (λ = 0.71073 Å)
Temperature: 100 K

Table S2. Connectivity in structure native-$\{Nb_6O_{19}\}$.

Label	Connection(s) to $\{Nb_6O_{19}\}$	Average M–O Bond Distance (Å)
1	Connect with O_t of $\{Nb_6O_{19}\}$	3.244(6)
2	Connect with O_b of $\{Nb_6O_{19}\}$	2.802<br>2.764(4)<br>2.613(4)
3	Connect with O_b of $\{Nb_6O_{19}\}$	3.243<br>3.080<br>2.741
4	Connect with O_b of $\{Nb_6O_{19}\}$	2.888<br>2.835(5)<br>2.935
5A	Coordinated with $\{Nb_6O_{19}\}$	N/A (H bond)
5B	Coordinated with $\{Nb_6O_{19}\}$	N/A (H bond)

Table S3. Connectivity in structure Mg-$\{Nb_6O_{19}\}$.

Label	Connection(s) to $\{Nb_6O_{19}\}$	Average M–O Bond Distance (Å)
1	Connect with O_t of $\{Nb_6O_{19}\}$	2.026(7)
2	Connect with O_t of $\{Nb_6O_{19}\}$	2.051(7)<br>2.056(7)
3	Connect with O_t of $\{Nb_6O_{19}\}$	2.011(9)
4	Connect with O_t of $\{Nb_6O_{19}\}$	2.089(10)<br>1.961(10)

Table S4. Connectivity in structure Li-$\{Nb_6O_{19}\}$.

Label	Connection(s) to $\{Nb_6O_{19}\}$	Average M–O Bond Distance (Å)
1–4	Coordinated with $\{Nb_6O_{19}\}$	N/A (H bond)

Table S5. Connectivity in structure Na-$\{Nb_6O_{19}\}$.

Label	Connection(s) to $\{Nb_6O_{19}\}$	Average M–O Bond Distance (Å)
1	Connect with O_t of $\{Nb_6O_{19}\}$	2.471<br>2.458
2	Connect with $\{Nb_6O_{19}\}$	N/A (H bond)
3	Connect with O_b of $\{Nb_6O_{19}\}$	2.484(2)<br>2.436<br>2.4365(17)

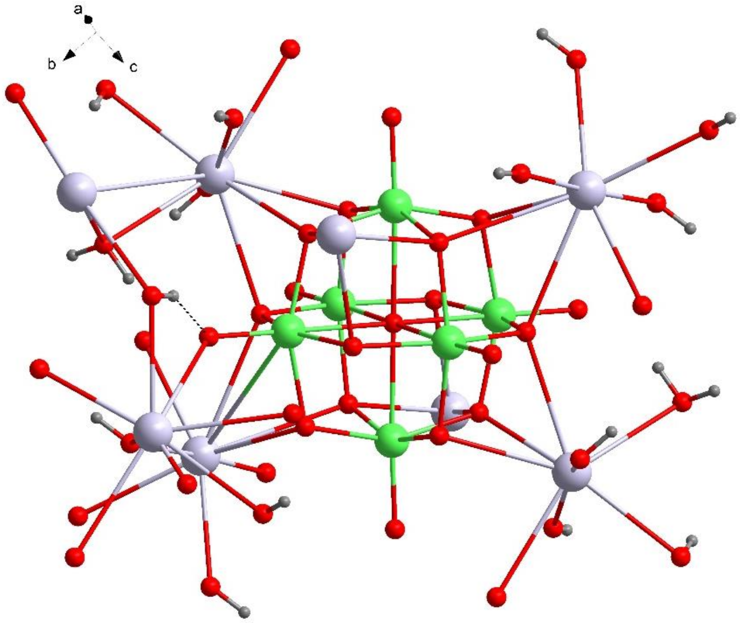

Figure S2. Ball-and-stick representation of native-PONb that highlights bond connections. Water omitted for clarity. Color code: Nb (green), O (red), K (purple), H (grey), H-bond (dash line).

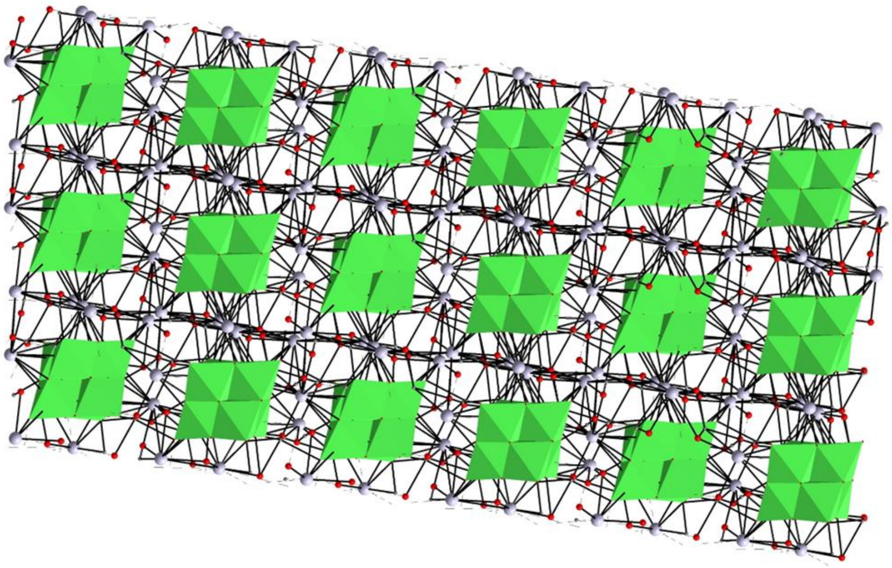

Figure S3. Native-PONb network with polyhedral representation in *ac*-plane. Water omitted for clarity. Color code: Nb polyhedral (green), O (red), K (purple), H (grey), H-bond (dash line).

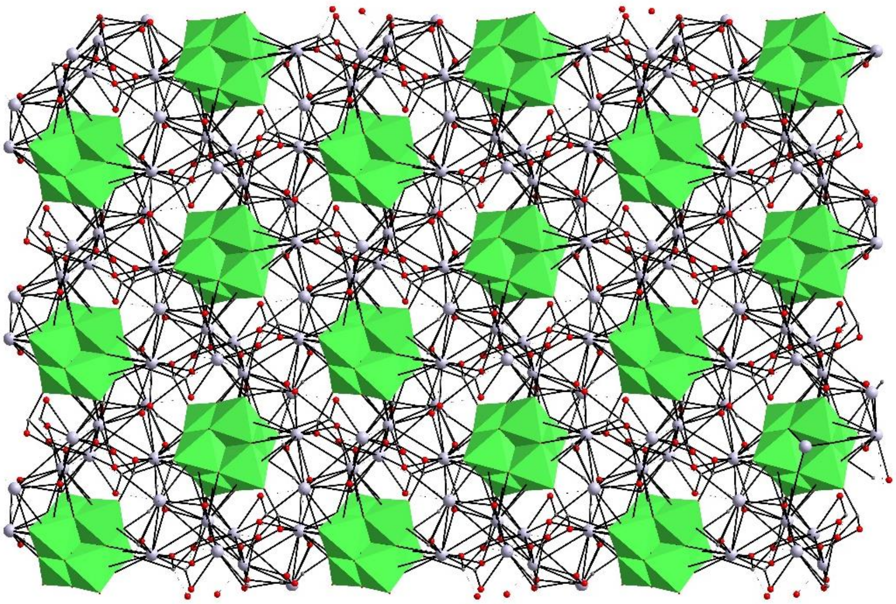

Figure S4. Native-PONb network with polyhedral representation in *bc*-plane. Water omitted for clarity. Color code: Nb polyhedral (green), O (red), K (purple), H (grey), H-bond (dash line).

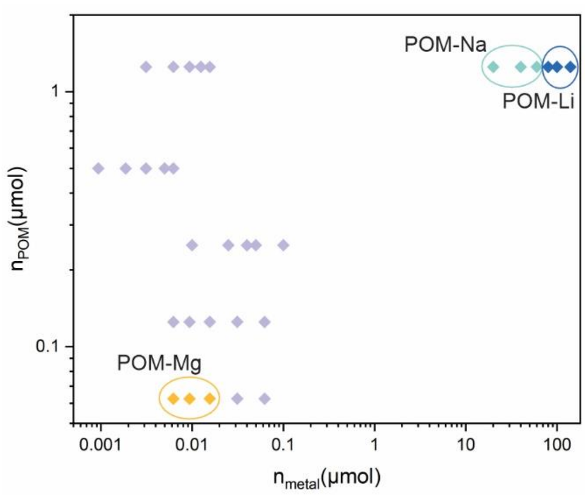

Figure S5. The specific conditions for single crystal growth, where successful formations of Li-PONb (blue), Na-PONb (green), Mg-PONb (yellow) are highlighted, while the purple markers indicate failed attempts to grow Mg-PONb crystals.

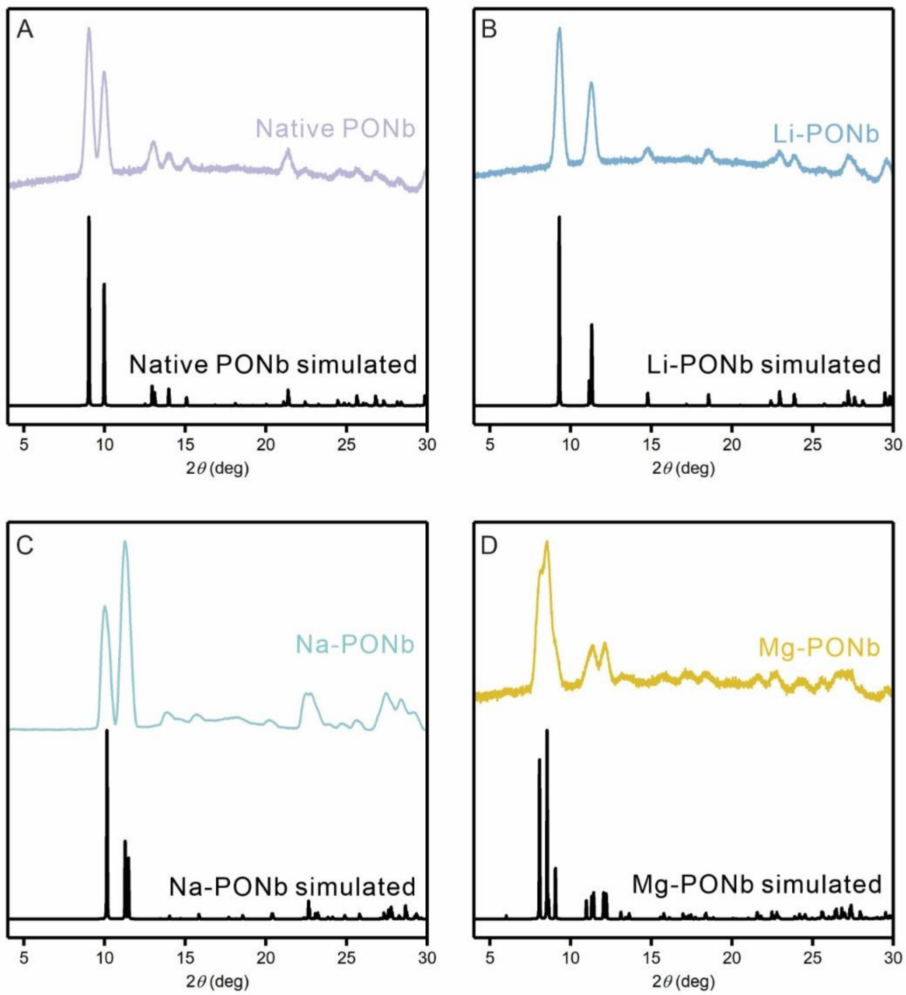

Figure S6. Powder X-ray diffraction patterns of (A) Native-PONb, (B) Li-PONb, (C) Na-PONb, and (D) Mg-PONb, measured using an oil-assisted method to preserve lattice water. Simulated patterns based on single-crystal data are shown at the bottom of each panel for comparison. The data demonstrate that all bulk samples retain structures consistent with their corresponding single crystals.

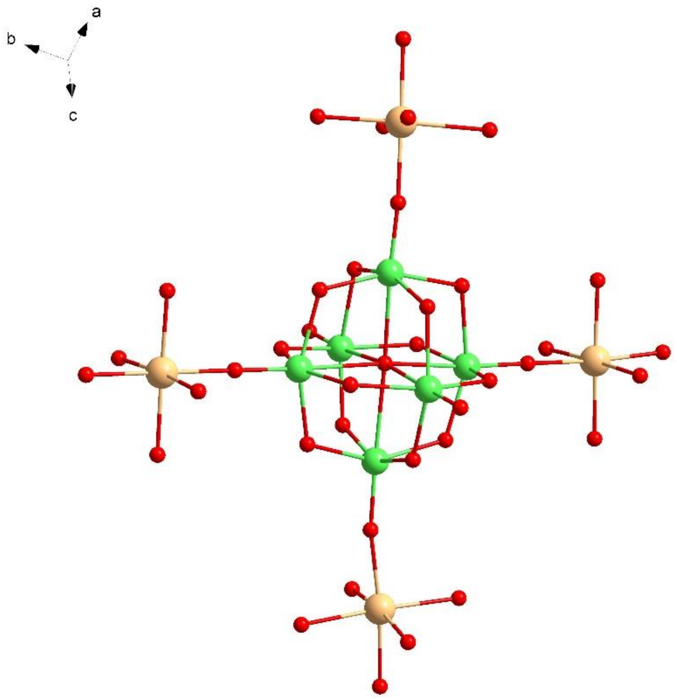

Figure S7. Ball-and-stick representation of Mg-PONb that highlights bond connections. Water omitted for clarity. Color code: Nb atom (green), O (red), Mg (yellow).

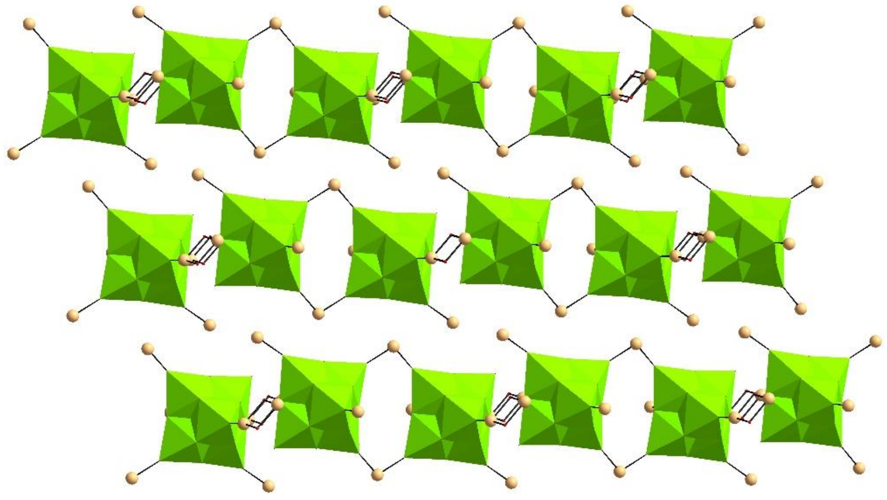

Figure S8. Mg-PONb network with polyhedral representation in *ac*-plane. Water omitted for clarity.   Color code: Nb polyhedral (green), O (red), Mg (yellow).

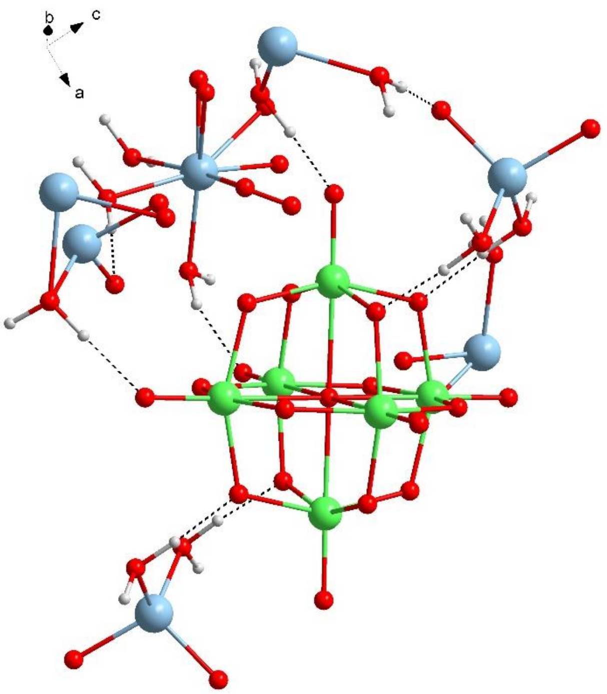

Figure S9. Ball-and-stick representation of Li-PONb that highlights bond connections. Water omitted for clarity. Color code: Nb atom (green), O (red), Li (blue), H (grey), H-bond (dash line).

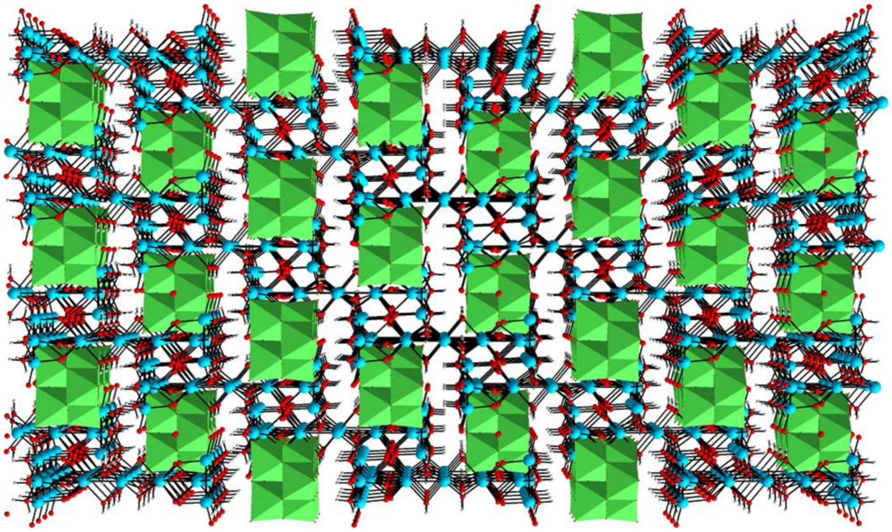

Figure S10. Li-PONb network with polyhedral representation in *ac*-plane. Water omitted for clarity. Color code: Nb polyhedral (green), O (red), Li (blue), H (grey), H-bond (dash line).

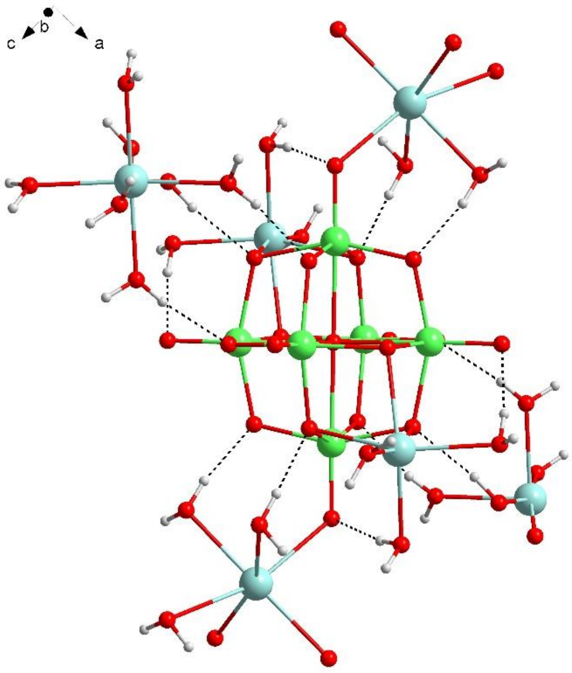

Figure S11. Ball-and-stick representation of Na-PONb that highlights bond connections. Water omitted for clarity. Color code: Nb atom (green), O (red), Na (light blue), H (grey), H-bond (dash line).

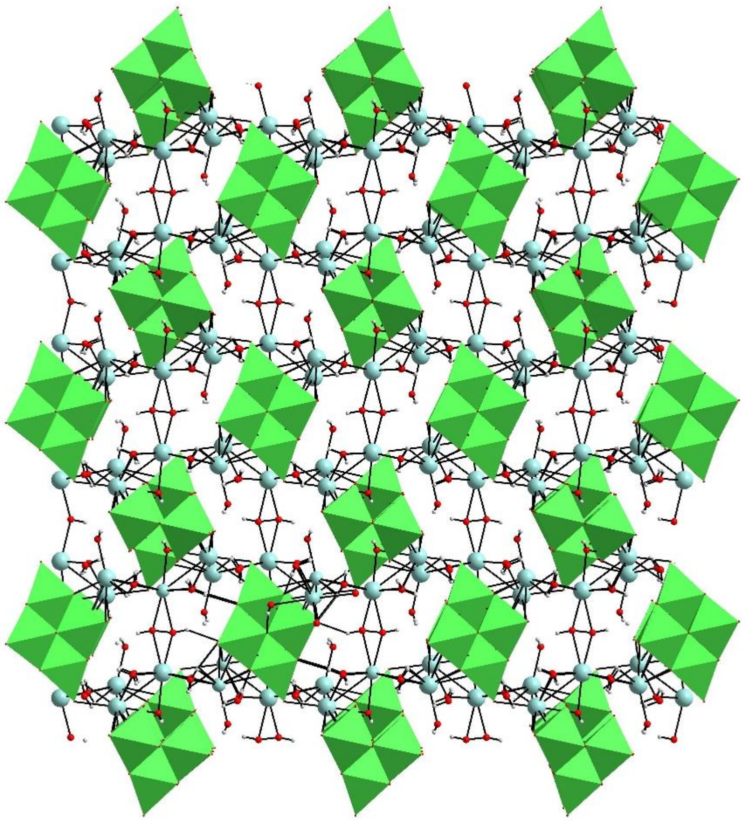

Figure S12. Na-PONb network with polyhedral representation in *ab*-plane. Water omitted for clarity. Color code: Nb polyhedral (green), O (red), Na (light blue), H (grey), H-bond (dash line).

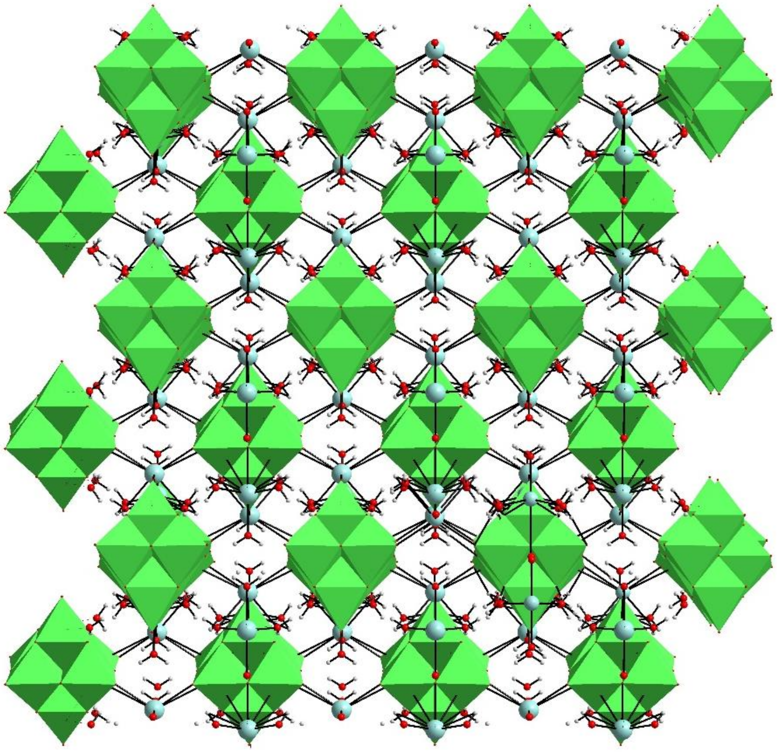

Figure S13. Na-PONb network with polyhedral representation in *bc*-plane. Water omitted for clarity. Color code: Nb polyhedral (green), O (red), Na (light blue), H (grey), H-bond (dash line).

Table S6. Summary of bonding parameters for M-PONb (M=K^+, Li^+, Na^+, Mg^{2+}) and their corresponding calculated binding energies.

	M^{n+}–O_t			M^{n+}–O_b			Number of O_t/O_b/H-bond
	Exp. Bond Length [Å]	Theor. Bond Length [Å]	Binding Energy (eV)	Exp. Bond Length [Å]	Theor. Bond Length [Å]	Binding Energy (eV)	
Li^+	N/A	3.12	–0.068	N/A	2.812	–0.013	0/0/7
Na^+	2.465	2.781	–0.463	2.452	2.796	–0.449	2/2/2
K^+	3.244	3.388	–0.237	2.948	2.789	–0.432	1/5/2
Mg^{2+}	2.034	2.316	–0.615	N/A	2.324	–0.547	4/0/0

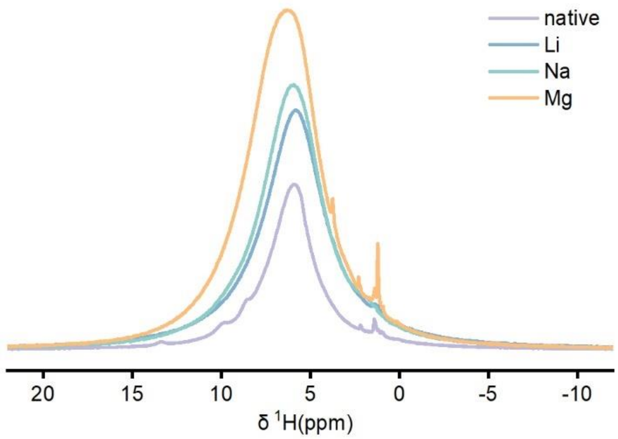

Figure S14. Quantitative ^{1}H spin (Hahn) echo solid-state NMR spectra of all four PONbs, showing a broad water resonance at 6 ppm and, in Native-PONb, three acidic O_bH resonances between 8-14 ppm. The weak, sharp peaks upfield of the water resonance are from an alcohol impurity.

Table S7: Relative ratios of water environments in PONbs. The first three columns show relative water content between PONbs normalized to Native-PONb, while the last column shows relative ratios of the two sites within each PONb. Site A is associated with H_2O–PONb interactions, and site B is associated with H_2O–M interactions (M = K^+, Mg^{2+}, Li^+, Na^+). Substitution of Mg, Li, or Na for K results in an increase in water content primarily due to an increase in site A content. Mg-PONb has the highest overall water content.

	Site A Integral	Site B Integral	Total water content	Site B: Site A ratio
Native-PONb	1.00	1.00	1.00	10.8:1
Mg-PONb	17.0	1.70	3.00	1.08:1
Li-PONb	8.58	1.44	2.04	1.80:1
Na-PONb	13.5	1.08	2.14	0.86:1

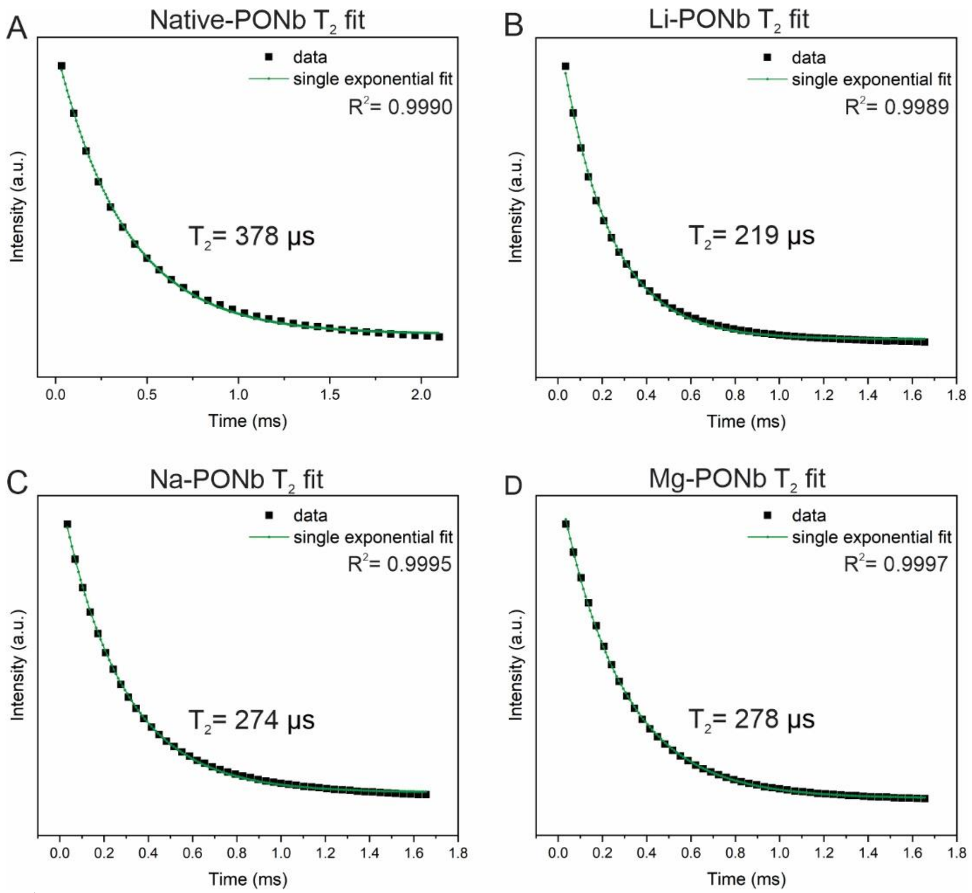

Figure S15: ^1H ssNMR T_2 relaxation measurements for all four PONbs used in the quantification of the ^1H signals. 48 spectra were obtained at different echo times, and total peak area of the broad line shape was used for fitting. Single-exponential fitting provided the best results, however the measured T_2 times are an average representation of water relaxation in the bulk sample due to the existence of multiple water sites.

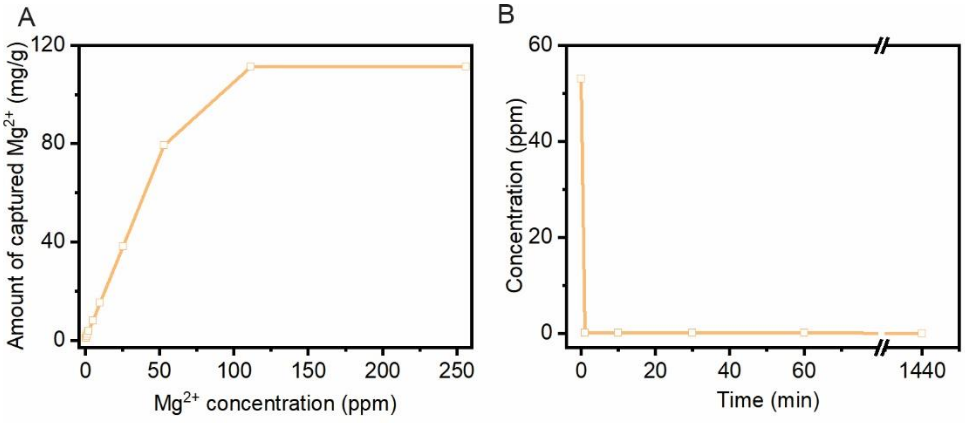

Figure S16. ICP-OES results Precipitation performances for a Mono-component Mg^{2+} Solution: (A) Experimental amount of captured per efficient amount of $\{Nb_6O_{19}\}^{8-}$. (B). Precipitation kinetics of Mg^{2+} by Native-PONb material over time. The plot illustrates a rapid decrease in Mg^{2+} concentration within the first 1 minute, reaching near-zero levels, followed by a stable concentration over the remaining time up to 1440 minutes.

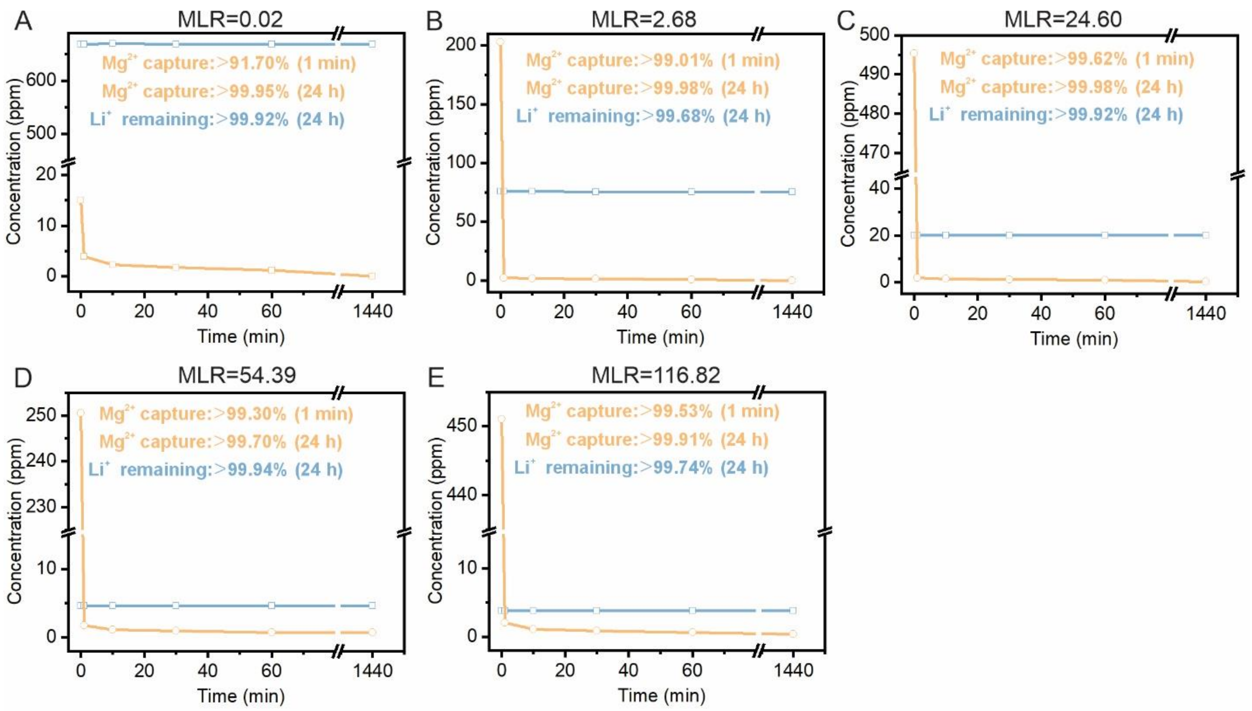

Figure S17. ICP-OES results for Li^+/Mg^{2+} separation performance of laboratory-prepared simulated brines mimicking major salt lakes with different MLR: (a) 0.02, (b) 2.68, (c)24.60, (d) 54.39, (e) 116.82. Yellow curve represents the variation in Mg^{2+} concentration, while the blue curve represents the variation in Li^+ concentration. The salt concentration of the binary salt mixture is less than 1000 ppm.

Table S8. The rejection of Li^+ and Mg^{2+} and Li^+/Mg^{2+} selectivity as a function of MLR of feed solution. The salt concentration of the binary metal salt mixture is less than 1000 ppm. $\mathbf{S_{Li/Mg}}$ represents Li^+/Mg^{2+} selectivity.

MLR	Feed (ppm)		Receive (ppm)		Li^+ rejection	Mg^{2+} rejection	$S_{Li/Mg}$
	Li^+	Mg^{2+}	Li^+	Mg^{2+}			
0.02	669.315	14.997	668.812	0.007	0.08%	99.95%	2050.09
0.82	4.754	3.885	4.732	0.001	0.48%	99.97%	3866.00
2.68	75.580	202.715	75.337	0.034	0.32%	99.98%	5872.21
3.47	6.954	24.141	6.921	0.004	0.47%	99.98%	5461.03
5.95	4.851	28.883	4.832	0.005	0.40%	99.98%	5753.36
17.17	5.096	87.517	5.078	0.056	0.37%	99.94%	1557.04
24.60	20.135	495.297	20.119	0.116	0.08%	99.98%	4267.04
35.84	4.620	165.591	4.619	0.037	0.02%	99.98%	4449.35
54.39	4.606	250.533	4.603	0.744	0.06%	99.70%	336.40
116.82	3.861	451.073	3.851	0.409	0.26%	99.91%	1101.29
200.18	2.444	489.233	2.442	0.507	0.09%	99.90%	964.32

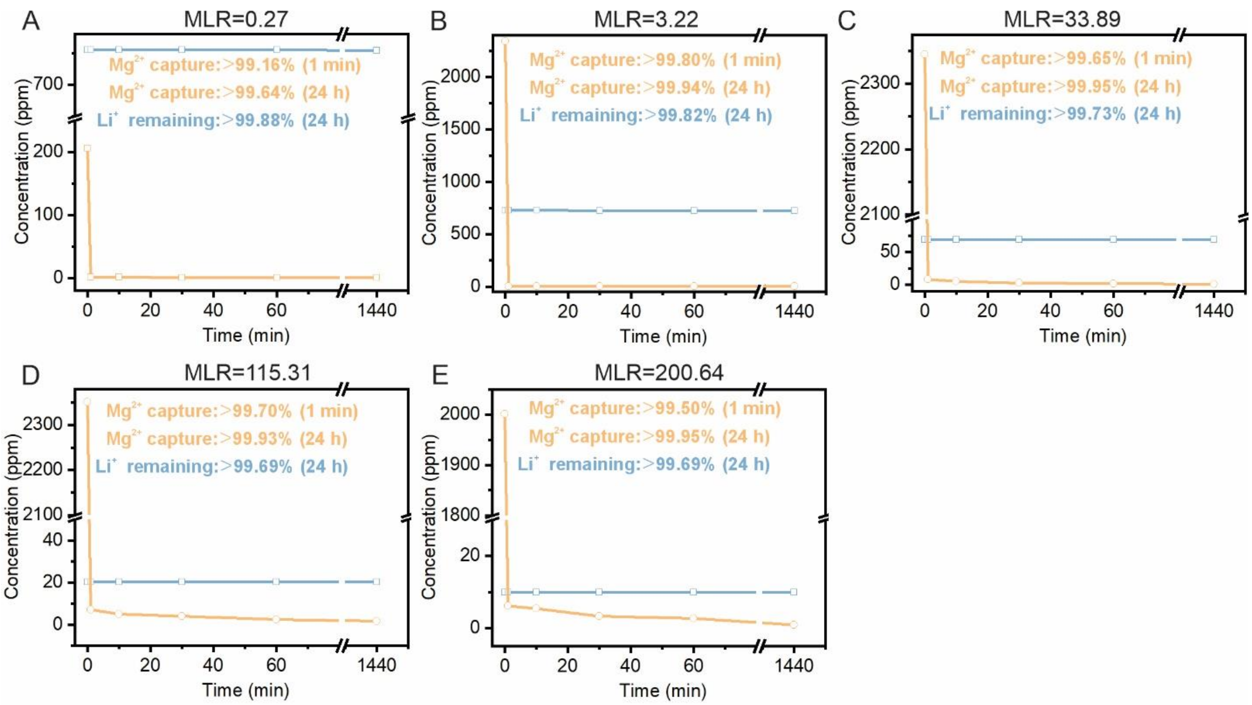

Figure S18. ICP-OES results for Li^+/Mg^{2+} separation performance of laboratory-prepared simulated brines mimicking major salt lakes with different MLR: (A) 0.27, (B) 3.22, (C)33.89, (D) 115.31, (E) 200.64. Yellow curve represents the variation in Mg^{2+} concentration, while the blue curve represents the variation in Li^+ concentration. The salt concentration of the binary metal salt mixture is between ~1000–3000 ppm.

Table S9. The rejection of Li^+ and Mg^{2+} and Li^+/Mg^{2+} selectivity as a function of MLR of feed solution. The salt concentration of the binary salt mixture is between ~1000–3000 ppm. $\mathbf{S_{Li/Mg}}$ represents Li^+/Mg^{2+} selectivity.

MLR	Feed(ppm)		Receive (ppm)		Li^+ rejection	Mg^{2+} rejection	$S_{Li/Mg}$
	Li^+	Mg^{2+}	Li^+	Mg^{2+}			
0.27	754.522	205.518	753.621	0.743	0.12%	99.64%	276.19
3.22	727.011	2341.691	725.718	1.381	0.18%	99.94%	1692.63
33.89	69.184	2344.732	68.994	1.093	0.27%	99.95%	2139.33
115.31	20.382	2350.151	20.319	1.602	0.31%	99.93%	1462.48
200.64	9.972	2000.748	9.941	0.976	0.31%	99.95%	2043.57

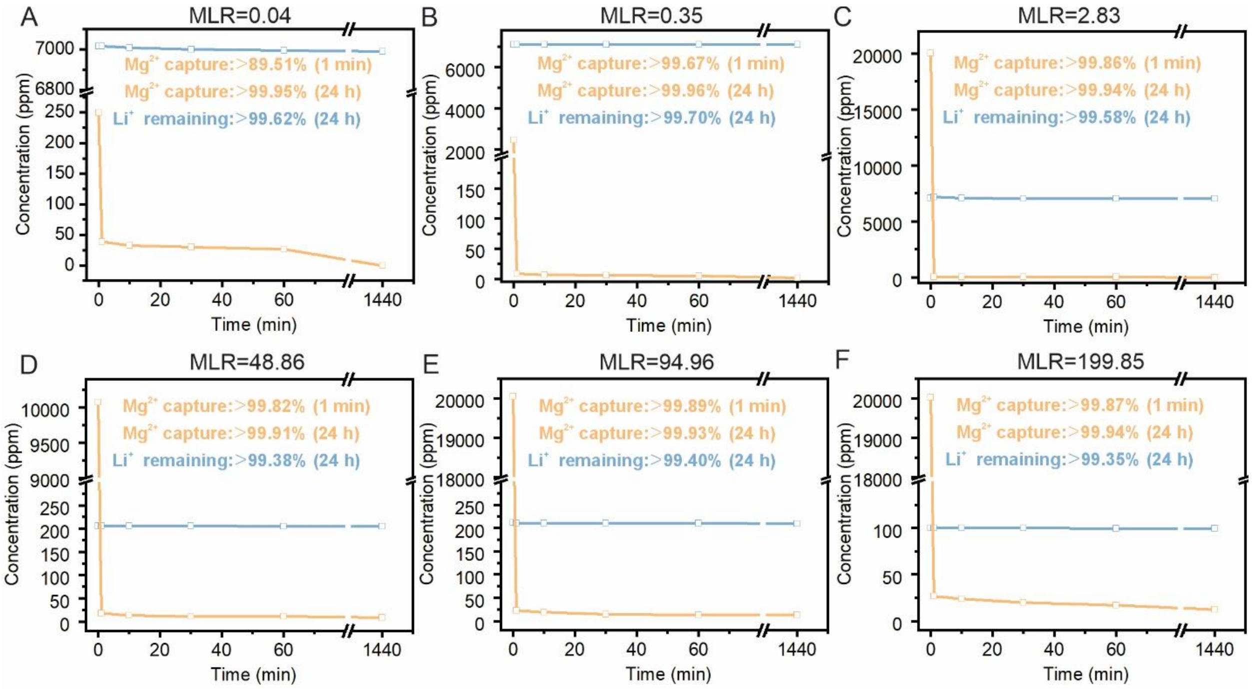

Figure S19. ICP-OES results for Li^+/Mg^{2+} separation performance of laboratory-prepared simulated brines mimicking major salt lakes with different MLR: (A) 0.04, (B) 0.35, (C)2.83, (D) 48.86, (E) 94.96, (F)199.85. Yellow curve represents the variation in Mg^{2+} concentration, while the blue curve represents the variation in Li^+ concentration. The salt concentration of the binary metal salt mixture is between ~7000–30000 ppm.

Table S10. The rejection of Li^+ and Mg^{2+} and Li^+/Mg^{2+} selectivity as a function of MLR of feed solution. The salt concentration of the binary salt mixture is between ~7000–30000 ppm. $\mathbf{S_{Li/Mg}}$ represents Li^+/Mg^{2+} selectivity.

MLR	Feed(ppm)		Receive (ppm)		Li^+ rejection	Mg^{2+} rejection	$S_{Li/Mg}$
	Li^+	Mg^{2+}	Li^+	Mg^{2+}			
0.036	7016.104	249.719	6989.112	0.134	0.38%	99.95%	1856.41
0.35	7107.702	2479.011	7086.382	1.010	0.30%	99.96%	2447.10
2.83	7079.069	20037.910	7049.014	11.129	0.42%	99.94%	1792.87
48.86	206.190	10074.962	204.904	8.601	0.62%	99.91%	1164.07
94.96	211.179	20053.373	209.919	13.906	0.60%	99.93%	1433.46
199.85	100.165	20018.179	99.510	12.710	0.65%	99.94%	1564.70

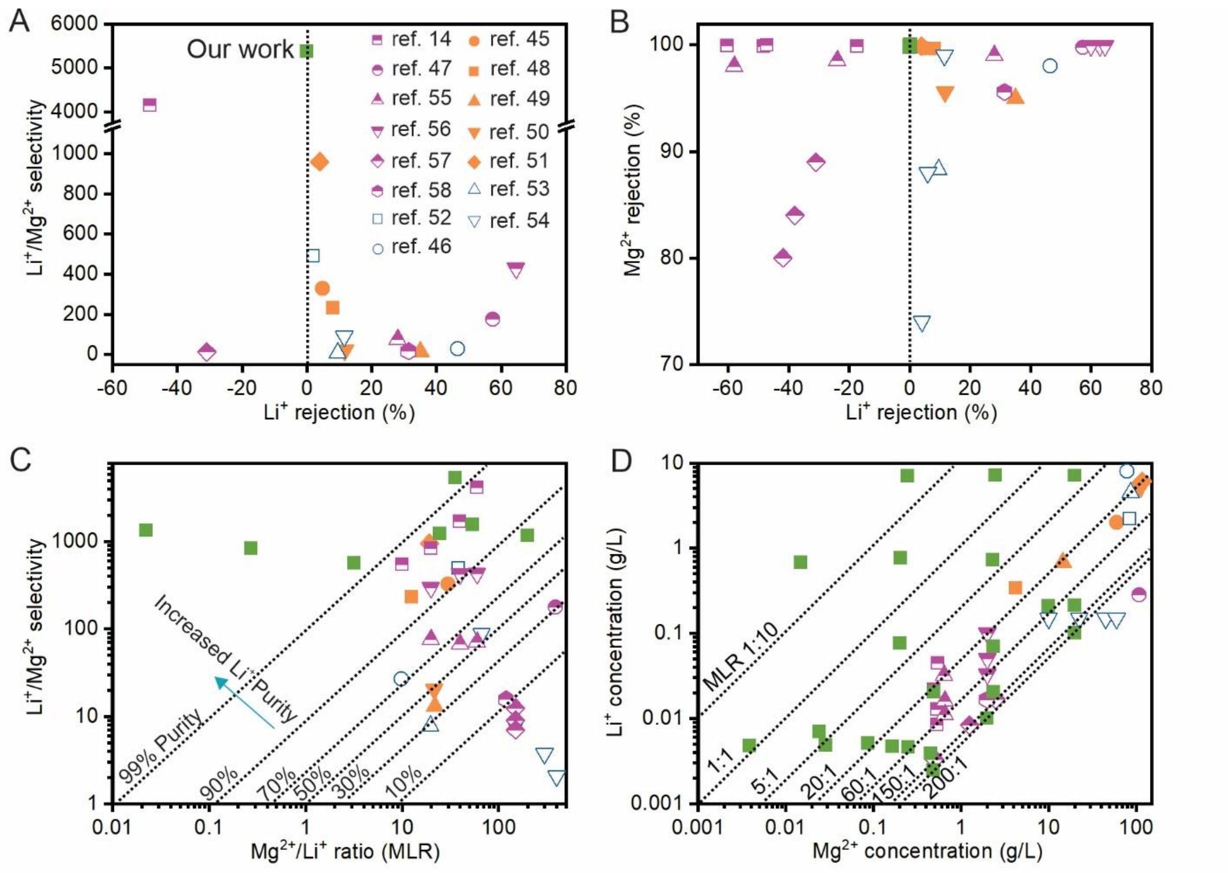

Figure S20. (A) Li^+/Mg^{2+} selectivity as a function of Li^+ rejection. Native-PONb materials exhibit the highest reported selectivity, exceeding precipitation methods by more than two orders of magnitude, while maintaining near-zero Li^+ rejection. In contrast, many membrane and electrochemical systems show significantly lower selectivity and suffer from positive or even negative Li^+ rejection. (B) Mg^{2+} rejection as a function of Li^+ rejection. Native-PONb achieves nearly 100% Mg^{2+} rejection with minimal Li^+ loss, significantly outperforming electrodialysis and membrane processes that exhibit clear trade-offs between Li^+ retention and Mg^{2+} removal. (C) Li^+/ Mg^{2+} selectivity as a function of Mg^{2+}/ Li^+ molar ratio (MLR), with permeated Li^+ purity indicated by dashed lines (ranging from 10% to 99%, calculated using **Equation (S3)** in the Supporting Information). Native-PONb maintains >90% Li^+ purity across a wide MLR range, reaching up to 99% at lower MLRs, far surpassing all reported systems. (D) Li^+ and Mg^{2+} concentrations of the feedwaters used in this study and in literature. Data points on the same dashed line have the same MLR. The broad range of conditions tested here (MLR = 0.02–200.63) represents the widest reported to date, underscoring the adaptability and robustness of Native-PONb materials for real-world brine applications**.** Additional data and comparative details are provided in **Tables S11**.

Table S11. Summary of the performance of Li^+/Mg^{2+} separation methods of the previously reported technologies.

Method	Key materials or technologies	MLR	Li^+ rejection	Mg^{2+} rejection	Mg^{2+} concentration (g/l)	Li^+ concentration (g/l)	Li^+/Mg^{2+} selectivity
**Precipitation**	Triammonium phosphate trihydrate(*45*)	30	4.88%	99.71%	60	2	328
	Hydrotalcite(*48*)	12.6	8.20%	99.60%	4.27	0.338	229.5
	Magnesium oxalate(*49*)	21.8	35%	95%	14.6	0.67	13
	TLPE(*50*)	21.49	11.70%	95.60%	112.4	5.23	20.07
	$MgAlCO_3$-LDHs(*51*)	19.22	3.91%	99.9%	117.06	6.09	960.90
**Electrodialysis**	EID-$MgNH_4PO_4$(*52*)	38.45	1.90%	99.80%	84.61	2.20	490.50
	S-ED process(*46*)	9.85	46.49%	98%	78.67	7.99	26.76
	constant-voltage ED(*53*)	19.68	9.50%	88.27%	87	4.42	7.72
	ED Membranes(*54*)	66.7	11.50%	99%	10	0.15	88.50
		150	6.00%	88%	22.5	0.15	7.83
		300	4.00%	74%	45	0.15	3.69
		400	3.00%	53%	60	0.15	2.06
**Membrane**	OSARIP(*14*)	10	-17.25%	99.790%	0.543	0.0444	548.00
		20	-48.34%	99.820%	0.497	0.0218	828.00
		40	-60.23%	99.910%	0.542	0.0128	1705.00
		60	-47.14%	99.960%	0.535	0.0084	4147.00
	(TBP)/$FeCl_3$+ PVC(*47*)	387.51	57.33%	99.74%	108.504	0.28	176
	PSS/PAH LBL(*55*)	20	28%	99%	0.645	0.032	75.00
		40	-24%	98.5%	0.655	0.016	67.00
		60	-58%	98%	0.655	0.011	70.00
	PSS/PAH NF(*56*)	20	63.00%	99.9%	2	0.1	295.00
		40	60.00%	99.9%	2	0.05	423.00
		60	64.60%	99.9%	2	0.033	430.00
	Positively charged surface(*57*)	150	-31%	89%	0.5	0.0033	12.37
		150	-38%	84%	1.25	0.00835	9
		150	-42%	80%	2.5	0.0167	7.00
	QEDTP(*58*)	120	31.36%	95.6%	1.9835	0.0165	15.60

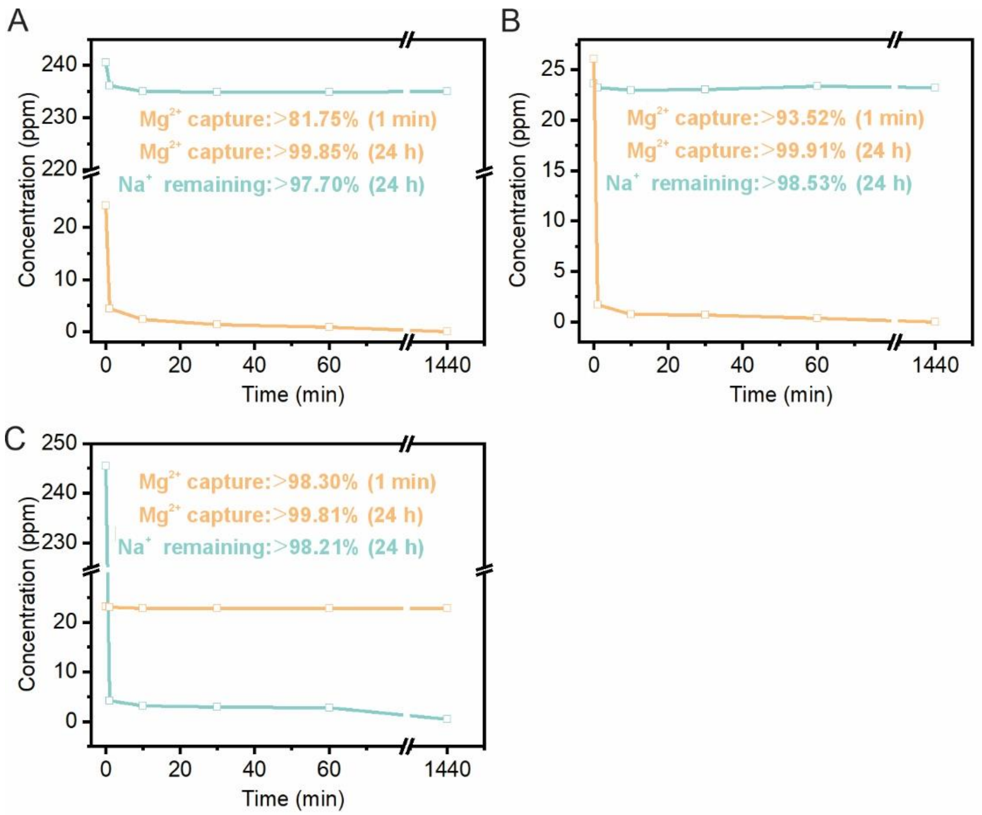

Figure S21. ICP-OES results for Na^{+}/Mg^{2+} separation performance of laboratory-prepared simulated brines mimicking major salt lakes with different Mg-Na-ratio: (A) 0.10, (B) 1.11, (C)10.59. Yellow curve represents the variation in Mg^{2+} concentration, while the green curve represents the variation in Na^{+} concentration. The salt concentration of the binary salt mixture is less than 300 ppm. When Na^{+} was introduced into the Mg^{2+} solution, at least 81.7% of Mg^{2+} was captured within one minute and over 99.8% Mg^{2+} was captured at the end of test, although 2.3% of Na^{+} was also captured over 24 hours. Despite this minor co-capture of Na^{+}, Native-PONb still demonstrated robust separation capabilities for Na^{+}/Mg^{2+}.

Table S12. The rejection of Na^{+} and Mg^{2+} and Na^{+}/Mg^{2+} selectivity as a function of Mg-Na ratios (MSR) of feed solution. The salt concentration of the binary metal salt mixture is less than 300 ppm. $S_{Na/Mg}$ represents Na^{+}/Mg^{2+} selectivity.

MSR	Feed(ppm)		Receive (ppm)		Na^{+} rejection	Mg^{2+} rejection	$S_{Na/Mg}$
	Na^{+}	Mg^{2+}	Na^{+}	Mg^{2+}			
0.10	240.540	24.224	234.996	0.038	2.30%	99.85%	630.42
1.11	23.593	26.088	23.246	0.025	1.47%	99.91%	1049.13
10.59	23.179	245.453	22.765	0.468	1.79%	99.81%	514.89

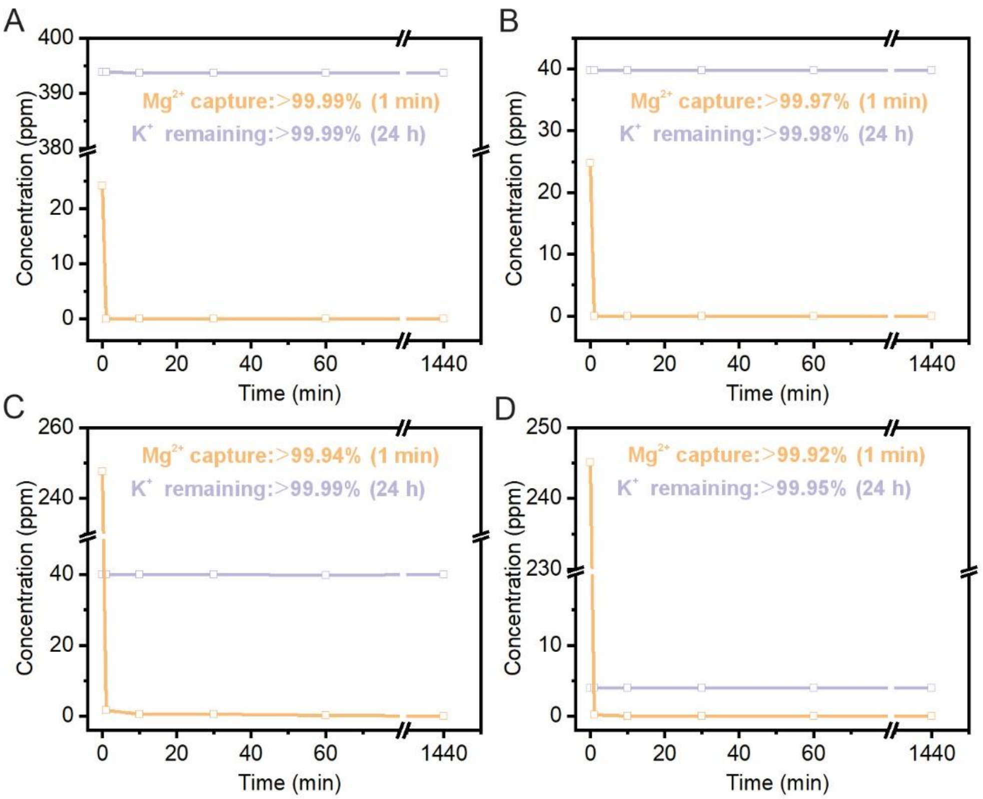

Figure S22. ICP-OES results for K^+/Mg^{2+} separation performance of laboratory-prepared simulated brines mimicking major salt lakes with different Mg-K-ratio: (A) 0.06, (B) 0.62, (C) 6.19, (D) 61.23. Yellow curve represents the variation in Mg^{2+} concentration, while the purple curve represents the variation in K^+ concentration. Due to the presence of K^+ ions within the Native-PONb material, the effective concentration of K^+ in the system is determined by subtracting the K^+ contribution from the Native-PONb from the total measured K^+ concentration. The results demonstrated that Native-PONb exhibits near-perfect separation efficiency for K^+/Mg^{2+}. Within one minute, over 99.4% of Mg^{2+} was captured, and 99.9% was captured within 10 minutes. After 24 hours, 99.9% of K^+ remained in the mixtures. These findings underscore the exceptional potential of Native-PONb materials for selective separation of K^+/Mg^{2+}.

Table S13. The rejection of K^+ and Mg^{2+} and K^+/Mg^{2+} selectivity as a function of Mg-K ratios (MPR) of feed solution. The salt concentration of the binary salt mixture is less than 500 ppm. $\mathbf{S_{K/Mg}}$ represents K^+/Mg^{2+} selectivity.

MPR	Feed(ppm)		Receive (ppm)		K^+ rejection	Mg^{2+} rejection	$S_{K/Mg}$
	K^+	Mg^{2+}	K^+	Mg^{2+}			
0.06	393.812	24.187	393.776	0.001	0.01%	99.99%	16231.48
0.62	39.800	24.706	39.791	0.008	0.02%	99.97%	2912.82
6.20	39.975	247.733	39.970	0.154	0.01%	99.94%	1608.48
61.23	4.003	245.133	4.001	0.193	0.05%	99.92%	1267.08

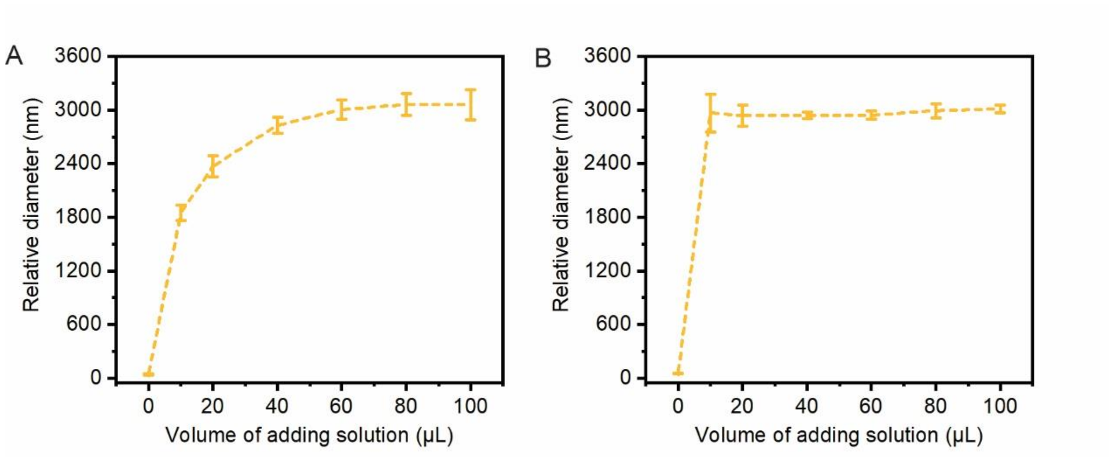

Figure S23. DLS studies on Native-PONb solution contacted with different concentrations of aqueous $MgCl_2$ solution: (A) 0.0125M, and (B) 0.025M. Tracking the assembly and precipitation of Mg-PONb phases.

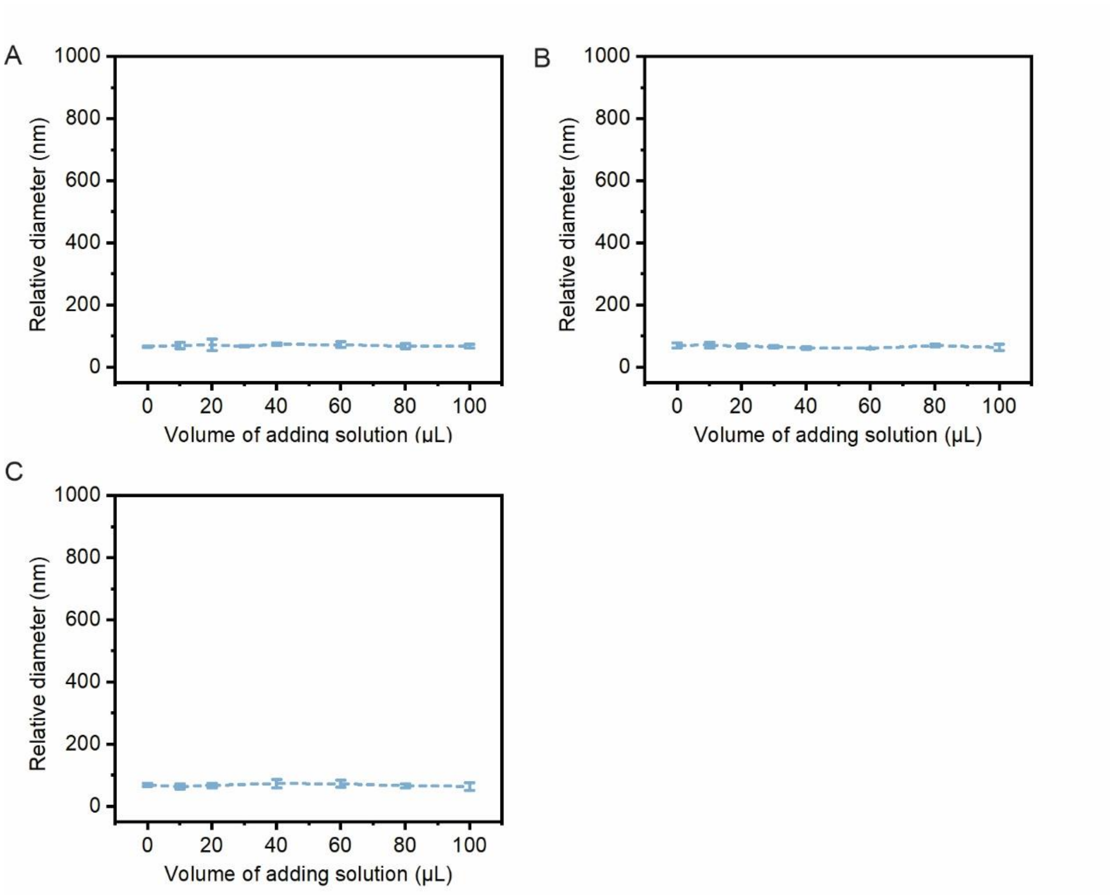

Figure S24. DLS studies on Native-PONb solution contacted with different concentrations of aqueous LiCl solution: (A) 0.0025 M, (B) 0.0125 M, and (C)0.025 M. Tracking the assembly and precipitation of Li-PONb phases.

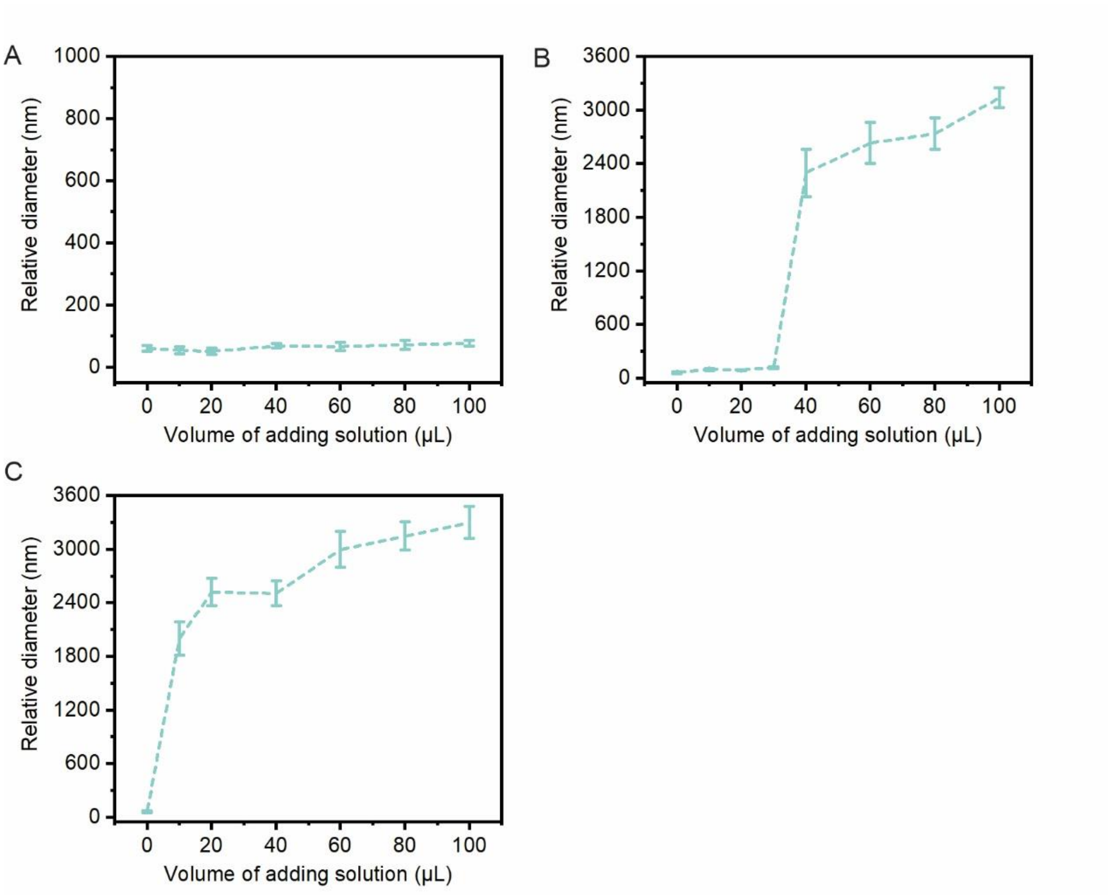

Figure S25. DLS studies on Native-PONb solution contacted with different concentrations of aqueous NaCl solution: (A) 0.10 M, (B) 0.50 M, and (C)1.0 M. Tracking the assembly and precipitation of Na-PONb phases.

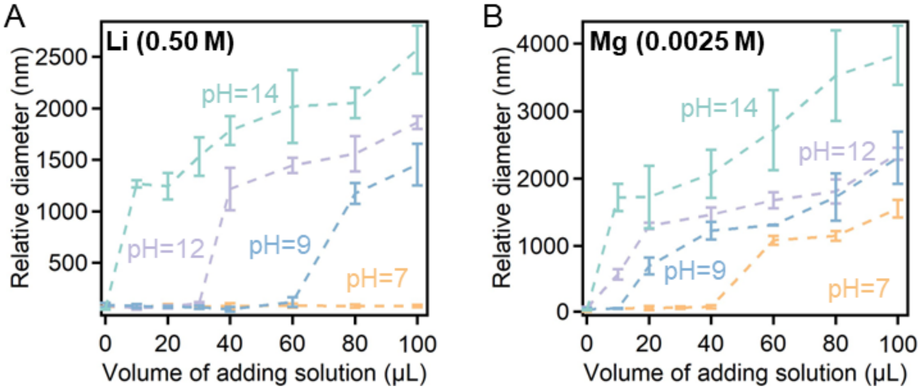

Figure S26. Effect of pH on the aggregation behavior of Native-PONb solutions upon addition of (A)LiCl and (B) $MgCl_2$, as monitored by DLS. Aggregation is defined as the appearance of particles larger than 1000 nm. Each curve represents a different pH condition adjusted by appropriate buffers or KOH.

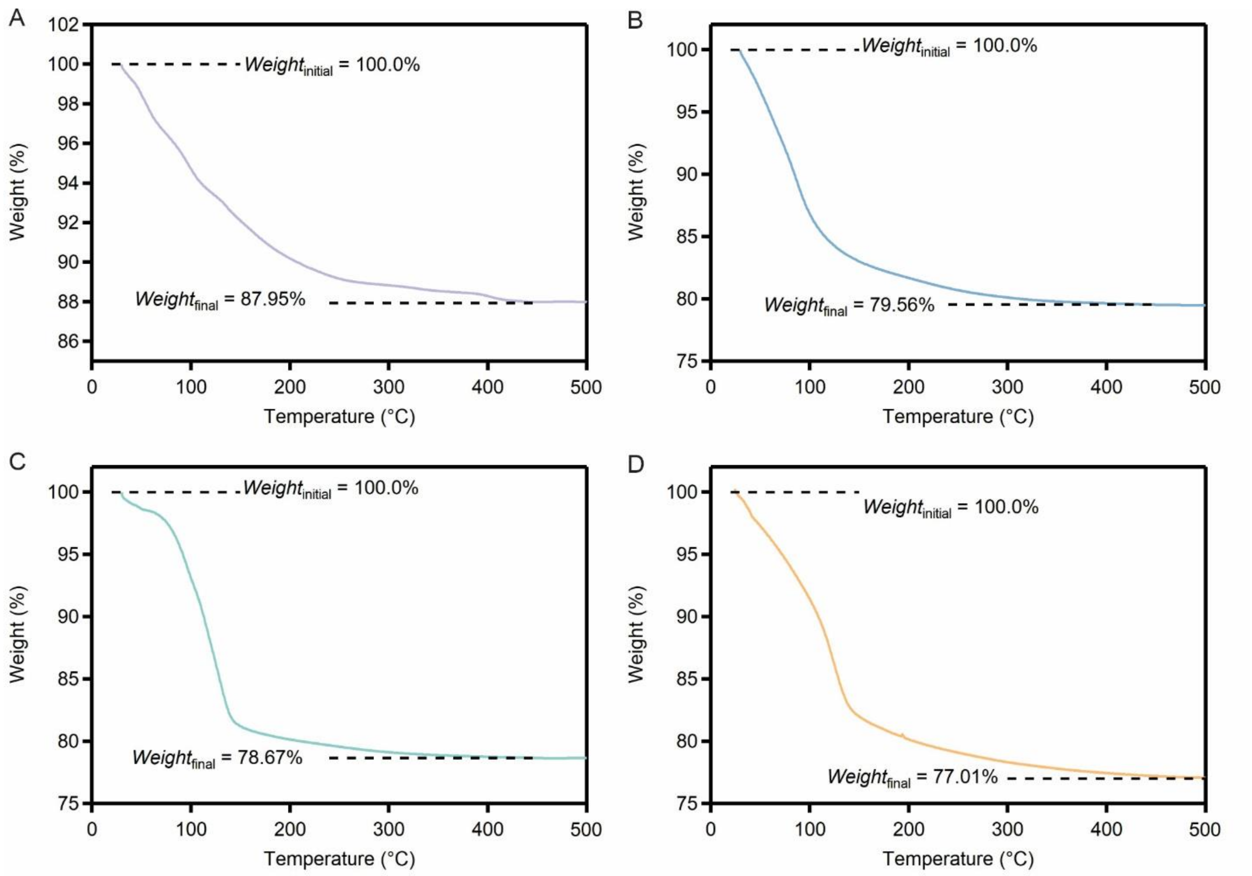

Figure S27. Thermogravimetric analysis of (A) Native-PONb, (B) Li-PONb, (C) Na-PONb, and (D) Mg-PONb. The loss of mass from room temperature to 500 °C was used to determine the water content of the crystals.

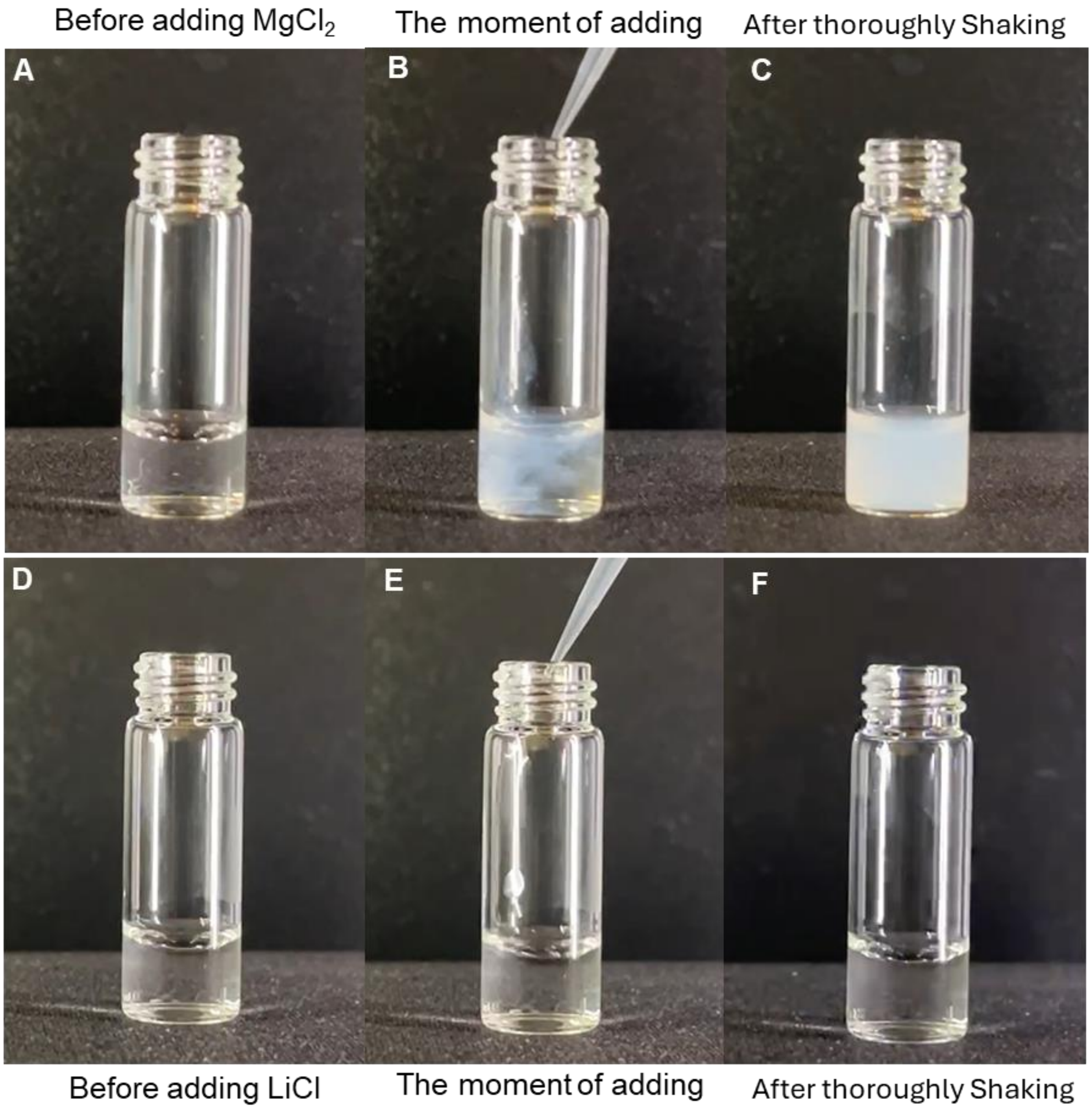

Figure S28. Photographs of Native-PONb (0.01M, 2 ml) solution interactions with aqueous (A-C) $MgCl_2$ (0.01 M, 50 μl) or (D-F) LiCl (2M, 50 μl) solutions. 2 ml of 0.01 M Native-PONb was placed in a glass vial, followed by the sequential addition of 50 μl of 2.0 M LiCl and 50 μl of 0.01 M $MgCl_2$. Upon the addition of $MgCl_2$, a significant amount of precipitate formed instantaneously, while the Li solution remained clear. This observation is consistent with the *in-situ* Raman spectroscopy results, providing robust evidence for the rapid and efficient separation mechanism.

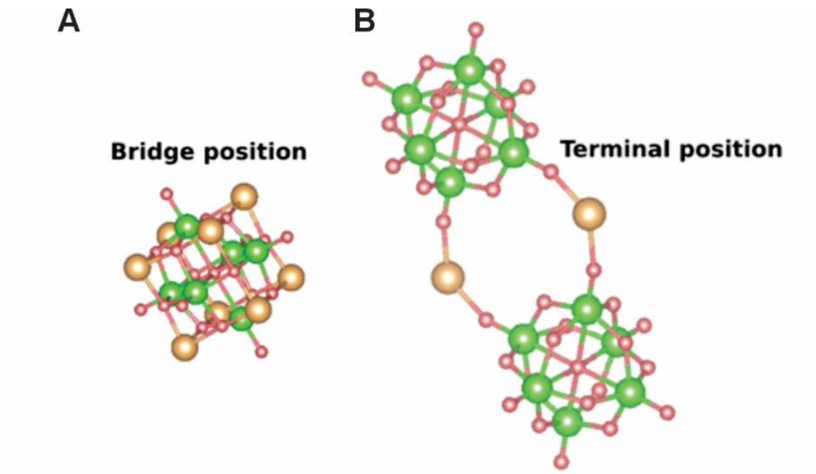

Figure S29. Different configurations for metal ions to connect with PONb. (A) represents the metal ions connect with bridge oxygen of PONb; (B) represents the metal ions connect with terminal oxygen of PONb.

Table S14. The rejection of Li^+ and Mg^{2+} and Li^+/Mg^{2+} selectivity as a function of MLR of feed solution for over five separation–regeneration cycles. The salt concentration of the binary metal salt mixture is less than 1000 ppm. $\mathbf{S_{Li/Mg}}$ represents Li^+/Mg^{2+} selectivity.

	**MLR**	**Feed(ppm)**		**receive (ppm)**		**Li^+ rejection**	**Mg^{2+} rejection**	$S_{Mg/Li}$
		**Li^+**	**Mg^{2+}**	**Li^+**	**Mg^{2+}**			
**Recycle1**	3.47	6.954	24.141	6.914	0.005	0.57%	99.98%	4800.57
	23.81	6.954	165.591	6.901	0.028	0.76%	99.98%	5869.06
	116.82	3.861	451.073	3.830	0.319	0.81%	99.93%	1402.57
	**Average**					0.71%	99.96%	4024.07
**Recycle2**	3.47	6.954	24.141	6.908	0.008	0.66%	99.97%	2997.75
	23.81	6.954	165.591	6.921	0.026	0.47%	99.98%	6338.84
	116.82	3.861	451.073	3.853	0.141	0.21%	99.97%	3192.25
	**Average**					0.45%	99.97%	4176.28
**Recycle3**	3.47	6.954	24.141	6.933	0.007	0.30%	99.97%	3438.40
	23.81	6.954	165.591	6.937	0.031	0.24%	99.98%	5328.74
	116.82	3.861	451.073	3.846	0.224	0.40%	99.95%	2005.76
	**Average**					0.31%	99.97%	3590.96
**Recycle4**	3.47	6.954	24.141	6.925	0.006	0.41%	99.98%	4006.84
	23.81	6.954	165.591	6.918	0.033	0.51%	99.98%	4992.08
	116.82	3.861	451.073	3.844	0.194	0.45%	99.96%	2314.72
	**Average**					0.46%	99.97%	3771.21
**Recycle5**	3.47	6.954	24.141	6.938	0.006	0.23%	99.98%	4014.36
	23.81	6.954	165.591	6.920	0.042	0.49%	99.97%	3923.48
	116.82	3.861	451.073	3.839	0.106	0.58%	99.98%	4230.86
	**Average**					0.43%	99.98%	4056.23

Table S15. Quantitative analysis of $Mg(OH)_2$ and PONb recovery during five regeneration cycles. The table lists the amount of Mg–PONb used in each cycle, the corresponding recovered $Mg(OH)_2$, and regenerated PONb. Both Mg^{2+} and PONb show consistently high recovery efficiencies across all cycles, supporting the regeneration results shown in **Figure 6d and 6e**.

	**Mg-PONb**		**$Mg(OH)_2$**			**PONb**		
	**mg**	**mmol**	**mg**	**mmol**	**R_{Mg}%**	**mg**	**mmol**	**R_{Nb}%**
**Recycle1**	500.11	0.401	92.18	1.581	98.50%	534.25	0.400	99.59%
**Recycle2**	420.94	0.338	78.29	1.342	99.39%	443.01	0.331	98.11%
**Recycle3**	320.85	0.257	59.23	1.016	98.65%	341.48	0.255	99.22%
**Recycle4**	220.65	0.177	40.87	0.701	98.99%	233.65	0.175	98.72%
**Recycle5**	163.51	0.131	30.22	0.518	98.77%	173.59	0.130	98.97%

Experimental Methods

Formula determination

Elemental analysis was conducted using inductively coupled plasma optical emission spectrometry (ICP-OES) on a Varian ICP-OES 720 Series to determine the total trace ions. The samples underwent digestion in 2 mL aqua regia solution, with sonication for 12 hours. Prior to ICP analysis, the samples were diluted with 2 wt% HNO_3. Standard solutions of niobium, potassium, lithium, sodium, and magnesium (Sigma Aldrich) were prepared in concentrations ranging from 1 to 500 ppm to create diluted standard solutions for calibration. Thermogravimetric analysis (TGA) was executed utilizing a TA Instruments Q5500 TGA-MS under an inert argon atmosphere. The sample temperature was increased at a controlled rate of 5 °C/min. The water content percentage was quantitatively assessed based on the mass reduction observed from 30 °C to 600 °C, with the values subsequently rounded to the nearest 0.5% H_2O for precision. Where appropriate, the experimental values are compared to literature values or to those calculated from the crystal structure (**Figure S27**).

N_2 adsorption isotherms and surface area measurements

Gas adsorption isotherms were obtained using an ASAP-2020 surface area analyzer. N_2 adsorption isotherms were recorded at 77 K within a liquid nitrogen bath. Prior to data acquisition, powder samples were degassed under vacuum at 120 °C for 12 hours.

X-ray photoelectron spectroscopy (XPS)

XPS analysis was conducted using a Thermo Scientific™ K-AlphaPlus™ instrument, equipped with a monochromatic Al Kα radiation source emitting at 1486.7 eV. The measurement area was configured to an elliptical shape of 200 × 400 μm, and a flood gun was employed for charge compensation. For spectral acquisition, the pass energy settings were 200 eV for survey spectra and 50 eV for high-resolution spectra, with an energy resolution maintained at 0.1 eV. The analysis chamber maintained the pressure of approximately 1×10^{-9} mbar during data acquisition. Data processing was executed using the Thermo Scientific Avantage XPS software, with peak fitting performed through a combination of Gaussian/Lorentzian shapes and a Shirley background subtraction. Reference for all peak positions was established using the C 1s peak from adventitious carbon at 284.4 eV. XPS spectra covered several binding energy regions, including Nb 3*d*, Mg 1*s*, C 1*s*, and O 1*s* orbitals, ensuring comprehensive surface composition analysis.

X-ray absorption spectra (XAS)

O K-edge X-ray absorption spectra were collected at the Advanced Light Source (ALS) at Lawrence Berkeley National Laboratory (Beamline 8.0.1.4). The measurements were performed using the medium-energy grating of the spherical grating monochromator of this undulator beamline under ultra-high vacuum condition of $\sim 10^{-9}$ Torr. Total-electron-yield mode was collected with a probe depth of less than 5 nm. Total-Fluorescence-yield signals were obtained through photon detection using channeltron detector, with a probe depth of approximately >100 nm. The photon energy scale was calibrated by measuring an XAS spectrum of TiO_2. All spectra were normalized to maximum peak intensity.

Raman spectroscopy

Raman spectroscopy measurements were conducted using two distinct systems. Non-*in-situ* Raman data were obtained with a Renishaw Qontor Raman microscope, which employed a 532 nm excitation laser (RL532-08, Renishaw). The spectra were collected using a 1800 l/mm grating, covering a range from 102 to 3700 cm^{-1}, with a laser power set at 10 mW. Subsequently, *operando* Raman spectroscopy was carried out on a confocal Raman spectrometer (HORIBA LabRAM HR Evolution) to elucidate the *in-situ* structural changes upon ion incorporation. The system employed a 532 nm laser with a power range of 1–3.2 mW, carefully chosen to optimize the signal-to-noise ratio while preserving the microstructure of the samples. Specifically, a 0.01 M Native-PONb solution (100 μl) was placed on a grooved slide for initial Raman spectral acquisition. Subsequently, 20 μl of 0.10 M $MgCl_2$ solution was sequentially added, with data collected at 5-second intervals for up to one minute. A distinct spectral shift from 889 cm^{-1} to 925 cm^{-1} was observed within 5 seconds, corresponding to the transition from v(Nb=O_t) to v(Nb-O_t-Mg). The absence of the 889 cm^{-1} peak indicates that the structural transition of Native-PONb to Mg-PONb was completed within 5 seconds (**Figure 4G**). Additionally, 20 μl of 1 M LiCl was similarly added to the 0.01 M Native-PONb solution on the grooved slide. *In-situ* Raman spectroscopy revealed that the peak at 889 cm^{-1} remained unchanged within one minute, confirming the absence of interaction with Li^+ within this timeframe (**Figure 4H**).

Dynamic light scattering measurement (DLS)

Dynamic light scattering (DLS) measurements were conducted using a Malvern Analytical Zetasizer Nano-ZS instrument. All samples were analyzed in a plastic cuvette at room temperature with a 633 nm laser source. The scattering angle was set at 173 degrees. The refractive index of the solute was 2.3403, and the refractive index of the solvent (water) was 1.33. Data analysis was performed using Zetasizer software version 7.13.

Generally, a 0.01M aqueous solution of Native-PONb (100 μl) was initially tested. Subsequently, different concentrations of LiCl, $MgCl_2$, and NaCl solutions (0.0025 M, 0.0125 M, 0.025 M, 0.10 M, 0.50M, 1.00 M) were added individually and measured (**Figure 4I, Figure S23 -S25**). The initial relative diameter of Native-PONb was approximately 59.95 nm, indicating no aggregation. Upon the addition of 0.10 M LiCl, the relative diameter exhibited no significant change, even with the addition of over 100 μL of LiCl. When the LiCl concentration was increased to 0.50 M, the relative diameter remained stable up to 30 μL of LiCl; however, with the addition of 40 μL, the relative diameter sharply increased to 1525 nm. This indicates that Li ions interact with Native-PONb, forming large aggregates. This rapid increase in diameter suggests the formation of a precipitate, corresponding to the solubility limit of the PONb aggregates. In contrast, when $MgCl_2$ was introduced, the addition of just 3 μL of 0.0025 M $MgCl_2$ caused the relative diameter to reach 578 nm. With the addition of 8 μL, the diameter exceeded 1000 nm, indicating that even a small amount of Mg ions can induce significant aggregation with PONb clusters. Higher concentrations of $MgCl_2$ were also tested: at 0.0125 M, the addition of 10 μL resulted in a relative diameter of 1852.83 nm, and at 0.025 M, the relative diameter dramatically increased to 2967.50 nm with the same volume addition (**Figure S23**). For comparison, similar concentrations of LiCl (0.0025 M, 0.0125 M, and 0.025 M) were tested, and in all cases, the relative diameter remained between 60–80 nm, indicating no significant aggregation (**Figure S24**). Similar tests with NaCl yielded results comparable to those of LiCl (**Figure S25**), with significant aggregation observed only when 40 μL of 0.5 M NaCl was added, resulting in an aggregate diameter of 2296.33 nm. These findings demonstrate that Native-PONb has a significantly lower solubility in the presence of Mg^{2+} ions compared to Li^+ and Na^+ ions. The high sensitivity of Native-PONb to Mg^{2+}, as evidenced by these experiments, aligns with the results obtained from ICP-OES.

Figure 4J shows the relationship between aggregate size and the molar ratios of Li^+, Mg^{2+}, and PONb. This figure was generated by smoothly connecting data points from the 0.0025 M $MgCl_2$ and 0.5 M LiCl curves in **Figure 4I**, which provided the corresponding volumes of $MgCl_2$ and LiCl solutions needed to form aggregates of various sizes, from 0 nm to 2000 nm. The plot displays the molar amounts of Li^+, Mg^{2+}, and PONb associated with each aggregate size, reflecting changes in ion composition as aggregate size increases and revealing characteristics of the aggregation dynamics in these solutions.

Solid-state nuclear magnetic resonance spectroscopy (ssNMR)

^1H ssNMR spectra were recorded at 9.4 T (400MHz for ^1H) using a Bruker BioSpin spectrometer equipped with an Avance IV NEO console and 1.3 mm double resonance HX magic angle spinning (MAS) probe. ^1H chemical shift was referenced with respect to tetramethylsilane (TMS) using the CH_2 resonance of adamantane as a secondary external reference at δ iso (^1H) = 1.85 ppm.

PONb samples were first dried in an oven for 48 hours at 80˚C. NMR samples were then packed in 1.3 mm zirconia rotors in air and closed with Vespel® SP1 drive caps. Packed sample masses for Native-PONb, -Mg, -Li, and -Na were 5.0 mg, 3.1 mg, 5.2 mg, and 4.6 mg, respectively. Samples were spun at $\boldsymbol{\nu}_R$ = 58-60 kHz at the magic angle using dry nitrogen to avoid moisture exposure. ^1H ssNMR spectra were obtained using a rotor-synchronized Hahn echo sequence (90°–τ–180°– τ–AQ, where τ is one rotor period and 2τ is the echo duration) with a 90° pulse of 1.4 μs. 64 scans were averaged using a repetition delay of 5 s (Mg-PONb and Li-PONb) or 10 s (Native-PONb and Na-PONb) which was long enough to reach full relaxation of all ^1H signals across all 4 samples.

T_2 relaxation measurements were obtained by varying the 2τ echo duration in rotor synchronized spin-echo experiment from 33.3 μs to 2.10 ms for the Native-PONb ($\boldsymbol{\nu}_R$ = 60 kHz) and 34.5 μs to 1.65 ms for the Mg-PONb, -Li, and -Na ($\boldsymbol{\nu}_R$= 58 kHz). For echo delay, signals were averaged out over 16 scans with a repetition time of 2 s. To ensure a constant initial steady condition prior to the recovery delay for each echo, a train of 50 evenly spaced (5 ms) 90° pulses (1.4 μs) were used. Peak area was fitted to the echo time with exponentials of the form $M_0 e^{-\frac{t}{T_2}} + c$ using the SciPy package in Python. All solid-state NMR data were acquired and processed using Bruker TopSpin 4.4.0. Fitting of T_2 relaxation data was performed in Python as described above, while spectral deconvolution was performed using the DMfit software(*59*).

Supplementary note 1: solid-state nuclear magnetic resonance spectroscopy

Quantitative ^1H spin-echo ssNMR measurements were performed under fast MAS ($\boldsymbol{\nu}_R \approx 60$ kHz). Na-PONb shows a weak and broad resonance around 2 ppm, which is associated with a basic hydroxyl (NbOH) site(*32, 42*). Native-PONb shows three low intensity resonances at 8.6, 9.9, and 13.4 ppm. Nyman et al.(*32*) has noted such acidic (downfield) ^1H resonances are associated with hydrogen bonding between bridging oxygens (O_b) in niobate clusters. Native-PONb and Mg-PONb show multiple weak but sharp signals between 1–4 ppm, indicative of highly mobile proton environments. These are associated with alcohol impurities(*32, 60*) from MeOH used during synthesis, and thus will not be discussed further. The water resonance provides the most information about the chemical structure of the PONbs. Its chemical shift (around 6 ppm in all four PONbs) is higher than that observed for physisorbed H_2O in zeolites (ranging from 3.5 to 4.9 ppm)(*61*). This shift is similar to that of H_2O in Brønsted acid sites in zeolites, indicating the highly charged nature of the niobate cluster(*43*). Notably, the water resonance in Mg-PONb was

slightly downfield compared to the other three PONbs, at 6.3 ppm vs 5.9, 5.8, and 6.0 ppm in Native-PONb, -Li, and -Na, respectively.

Quantification of water content in these four PONb materials is achieved by recording ^1H ssNMR echo that are fully T_1 relaxed with back-calculation of T_2 losses during the echo delay (~34 µs) knowing T_2 values of each sample (**see Figure S15**). T_2 values of 378 µs, 278 µs, 219 µs,
and 274 µs for Native-PONb, Mg-, Li-, and Na-, respectively correspond to T_2 losses of 8.4%, 11.7%, 14.6%, and 11.8%. The presence of multiple non-H_2O proton sites requires spectral deconvolution for accurate quantification of water content. Additionally, the broadening of the water resonance caused by dipolar coupling interactions of ^1H spins obscures the presence of multiple distinct water resonances. After normalization by number of scans, sample loading weight, anhydrous molecular weight, and T_2 losses, the ^1H line shape was deconvoluted, verifying its limited resolution. In all four samples, a single site deconvolution of the broad water resonance led to a poor fit and at least two components were needed to obtain a satisfactory deconvolution. One site, A, maintains its chemical shift across all four PONbs at 5.7 ppm. While in Native-PONb and -Li, site B was observed at 6.1 ppm, in Mg-PONb and -Na, the site shifted to 7.4 ppm and 6.7 ppm, respectively. This matches the observation from Raman spectroscopy, which suggests the existence of an Nb–O–M (M = Mg^{2+}, Na^+) bridging bond for Mg-PONb and -Na only. For these reasons, site A was associated with interactions between H_2O and the Nb metal center, while site B was associated with interactions between H_2O and M (M = K^+, Mg^{2+}, Li^+, Na^+). These sites A and B are designated as H_2O–Nb and H_2O–M interactions.

Structure determination

Single crystal X-ray diffraction studies for compounds 1-4 were performed using a Bruker D8 Venture diffractometer equipped with Mo K_α radiation (λ= 0.71073 Å). Crystals were mounted on a Cryoloop with Paratone oil. Data were collected in a nitrogen gas stream at 100(2) K using and scans. The data were integrated using the Bruker SAINT software program and scaled using the SADABS software program. Solution by direct methods (SHELXT(*62*)) produced a complete phasing model consistent with the proposed structure. All nonhydrogen atoms were refined anisotropically by full-matrix least-squares (SHELXL-2014).(*62*)

In the crystal structure of Na-PONb, hydrogen atoms on oxygen atoms were found in difference Fourier map and O-H bond distances were restrained to 0.85 Å. For highly disordered solvent molecules, the PLATON routine SQUEEZE(*63*) was used to account for the corresponding electrons as a diffuse contribution to the overall scattering without specific atom positions.

There is a disorder of Li and O atoms in Li-PONb structures, the ratio of two possible positions is 50:50. Atom O9 represents a disordered solvent, it is most probably MeOH or MeOH/water mixture, but it is not possible to properly model such solvent disorder.

In the case of Native-PONb, K5 and one of the bonded O atoms is disordered in two positions with a ratio of occupancy approximately 9:1. Atom K1 is disordered in two positions due to symmetry, the occupancy of K1 and bonded atom O20 was refined freely. Hydrogen atoms on oxygen atoms were found in difference Fourier map and O-H bond distances were restrained to 0.85 Å.

In Mg-PONb, part of the terminal O atoms bonded to Mg are disordered in two positions with occupancy fixed to 0.5. The crystal quality was lower in this case, we could see some of the hydrogen atoms on oxygen atoms in difference Fourier map, but not every position could be estimated. Therefore, we decided not to include hydrogen atoms in refinement.

In all cases, all atoms were refined based on the electron density and $\{Nb_6O_{19}\}^{8-}$ structural chemistry, and referenced to ICP-OES elemental analysis results with any remaining negative charge compensated by protons

Separation efficiency evaluation experiment

Precipitation performances for a Mono-component Mg^{2+} Solution

Approximately 3.87 mg of Native-PONb material was individually introduced into conical tubes containing 5.0 mL of $MgCl_2$ solutions with varying magnesium concentrations of 0.67, 1.18, 1.68, 2.50, 5.33, 10.29, 25.62, 53.09, 111.39, and 256.13 ppm. These concentrations were verified using ICP-OES. The mixtures were placed on a Cole-Parmer digital tube roller shaker to ensure continuous mixing at a speed of 1000 rpm with a 360°rotation for a duration of 1 minute and then left undisturbed for 24 hours. After the 24-hour period, the samples were subjected to centrifugation for 1 minute to achieve homogeneous suspensions. The supernatants were collected and analyzed for magnesium content using ICP-OES. The magnesium precipitation performance of Native-PONb was calculated by deducting the residual magnesium concentration from the initial concentration in the solution. In **Figure S16A**, the experimental amount of Mg^{2+} captured per efficient amount of $\{Nb_6O_{19}\}^{8-}$ as a function of Mg^{2+} concentration. The plot shows a rapid increase in the amount of Mg^{2+} captured up to a concentration of approximately 111 ppm, after which the captured amount levels off, indicating saturation

The precipitation kinetics of Mg^{2+} by Native-PONb material over time were tested using a similar method. The same mass of Native-PONb material (3.87 mg) was individually added to conical tubes containing 5.0 ml of $MgCl_2$ solutions with magnesium concentrations of 53.09 ppm. The mixtures were placed on a Cole-Parmer digital tube roller shaker to ensure continuous mixing at a speed of 1000 rpm with a 360°rotation for a duration of 10 seconds and then left undisturbed. At 1, 10, 30, and 60 minutes, as well as 24 hours, the samples were subjected to centrifugation for 1 minute at 5000 rpm (with a 30-second centrifugation for the 1-minute sample) to achieve homogeneous suspensions. The supernatants were collected and analyzed for magnesium content using ICP-OES. The magnesium precipitation performance of Native-PONb material was calculated by deducting the residual magnesium concentration from the initial concentration in the solution. In **Figure S16B**, the precipitation kinetics of Mg^{2+} by Native-PONb material over time. The plot illustrates a rapid decrease in Mg^{2+} concentration within the first 1 minute, reaching near-zero levels, followed by a stable concentration over the remaining time up to 1440 minutes. This indicates that the Native-PONb material has a rapid and strong capture capability for Mg^{2+}.

Separation performances of bimetal mimic brine solution

The separation performance of bimetallic mimic brine solutions by Native-PONb material over time was tested using a similar method. Binary salt mixtures of $MgCl_2$/LiCl, $MgCl_2$/NaCl, and $MgCl_2$/KCl with various concentrations and mass ratios were used as feed solutions. The detailed concentrations and mass ratios are shown in **Tables S8-S10, S12-S13**. Approximately 8.0 mg of Native-PONb material was individually added to conical tubes containing 1.0 ml of bimetal solutions (Mg^{2+} concentration was between 100–500 ppm). The mixtures were placed on a Cole-Parmer digital tube roller shaker to ensure continuous mixing at a speed of 1000 rpm with 360° rotation for 10 seconds, then left undisturbed. At 1, 10, 30, and 60 minutes, as well as 24 hours, the samples were centrifuged for 1 minute at 5000 rpm (with a 30-second centrifugation for the 1-minute sample) to achieve homogeneous suspensions. The supernatants were collected and analyzed for magnesium content using ICP-OES.

When the Mg^{2+} concentration was less than 100 ppm, approximately 4.0 mg of Native-PONb material was added to conical tubes containing 1.0 ml of bimetal solutions. When the Mg^{2+} concentration was between 1000–3000 ppm, approximately 8.0 mg of Native-PONb material was added to conical tubes containing 0.2 ml of bimetal solutions. When the Mg^{2+} concentration was over 7000 ppm, approximately 80.0 mg of Native-PONb material was added to conical tubes containing 0.2 ml of bimetal solutions. The magnesium precipitation performance of the Native-PONb material was calculated by deducting the residual magnesium concentration from the initial concentration in the solution.

To evaluate the separation performances, some equations were employed:
The salt rejection, R (%), is calculated according to Eq. (1):

$$R=\left(1-\frac{C_r}{C_f}\right)\times 100\% \qquad \text{(S1)}$$

where C_r and C_f is used to denote receive and feed solution concentrations, respectively, which is determined by ICP-OES.

The Li^+/Mg^{2+} selectivity, $S_{Li/Mg}$, could be calculated through the rejection of Li^+ (R_{Li}) and Mg^{2+} (R_{Mg}):

$$S_{Li/Mg}=\frac{1-R_{Li}}{1-R_{Mg}} \qquad \text{(S2)}$$

This formula can also be extended to $S_{Na/Mg}$ and $S_{K/Mg}$ by replacing R_{Li} in Formula 2 with R_{Na} and R_K, respectively.

As the purpose of the separation is to extract Li from a Li/Mg mixture in a simplified scenario with only Li^+ and Mg^{2+} cations, the purity, η_{Li}(*19*), is defined as the mass fraction of cations in the received solution that are Li^+:

$$\eta_{Li} \equiv \frac{C_{r,Li}}{C_{r,Li}+C_{r,Mg}} \qquad \text{(S3)}$$

The promising Li^+/Mg^{2+} separation performance of Native-PONb characterized by rapid separation within seconds, a broad operational range, and high efficiency, is driven by their unique structural and electrostatic properties. Native-PONb exhibits a highest negative charge density on their surface oxygen atoms, which results from Nb's relatively low electronegativity(*28, 31, 64*). This weaker electron-withdrawing ability enhances the nucleophilicity of the oxygen atoms, making them more available for cation coordination. Additionally, the longer M-O bond lengths and minimal Nb *d*-orbital participation in Native-PONb further increase accessibility to cations(*28, 65*). Mg^{2+}, with its higher charge density and small ionic radius, demonstrates stronger electrostatic interactions with the negatively charged PONb surface due to its high charge-to-radius ratio (z/r)(*66*). This interaction is further strengthened by the formation of shorter Mg-O bonds at the terminal oxygen sites, which are associated with lower binding energies. These shorter bonds not only stabilize the Mg^{2+} coordination but also promote the self-assembly of Mg-PONb complexes into large porous structures with significantly higher surface areas. In contrast, Li^+, with its lower charge density, is heavily solvated in solution, surrounded by a robust solvation shell that diminishes its direct interaction with the PONb surface, significantly reducing its affinity for coordination(*67*). This combination of strong Mg^{2+} attraction and limited Li^+ interaction underpins the exceptional efficiency and selectivity observed in the Li^+/Mg^{2+} separation process using Native-PONb.

Molecular Dynamics Simulations

Molecular dynamics (MD) simulations were conducted using the Large-scale Atomic/ Molecular Massively Parallel Simulator (LAMMPS)(*68*) to investigate the behavior of PONb in aqueous solution. The simulations utilized the Lennard-Jones (LJ) force field(*69*) with long-range Coulombic interactions to efficiently model atomic interactions. The TIP3P(*70*) model was employed to describe water molecules, providing a simplified yet effective approach for simulating their behavior across various environments. Lorentz-Berthelot rules(*71*) (S4) were used to calculate the LJ parameters for species i and j in the studied system.

$$\varepsilon_{ij} = \sqrt{\varepsilon_i \varepsilon_j} \quad , \quad \sigma_{ij} = \left(\sigma_i + \sigma_j\right)/2$$

(S4)

The simulation box dimensions were set to 160 Å in each direction, containing 125,000 water molecules(*72*). The PONb molecules were randomly placed within the water box. Periodic boundary conditions were applied in all three dimensions to mimic an infinite system. The particle-particle particle-mesh (PPPM)(*73*) method was used to efficiently handle long-range electrostatic interactions with a precision of 10^{-5}. The system was equilibrated at 300 K using the NVT ensemble, followed by production runs in the NPT ensemble to ensure proper density and pressure conditions. The simulation timestep was set to 2 fs, and the optimization time was approximately 40 ns. After reaching equilibrium, Li^+, Mg^{2+}, Na^+, and Cl^- ions were added to the simulation box. The concentrations of cations and anions were adjusted to ensure electrical neutrality of the solution. To ensure sufficient reaction and contact between PONb and metal ions, following simulations were performed at least 60 ns under NVT ensemble.

Figure 4A shows the equilibrium configuration of PONb molecules in aqueous solution, that is, the initial configuration before the addition of different metal cations. At this stage, PONbs were relatively isolated and remained relatively stable, with no obvious aggregation tendency. Thereafter, several different ion sets were added to the simulation box to study the interactions between PONbs and different metal ions, as well as the aggregation state between PONb molecules. Here, four configurations were studied: the first configuration is Li^+ ions, the second is a mixture of Li^+ and Mg^{2+} ions with a ratio of 10:1, the third is a one-to-ten mixture of Li^+ and Mg^{2+} ions, and the last is pure Mg^{2+} ions. **Figures 4C-F** show the MD snapshots after 60 ns of the addition of different metal ions. **Figure 4C** shows the snapshot after the addition of Li^+ ions. The aggregation state of PONb was basically the same as the initial configuration (**Figure 4A**) with no PONb clusters. Some Li^+ ions were adsorbed on PONB, but most Li^+ ions were still scattered in the aqueous solution. The MD snapshots after adding a mixture of Mg^{2+} and Li^+ ions were shown in **Figure 4D and E**. The PONb molecules were clustered together and adsorbed almost all Mg^{2+} ions independent of the concentration. However, the interaction between PONb and Li^+ ions was relatively weak, which was manifested by less than 5% Li^+ ion adsorption and more than 95% of free Li^+ ions even in a Li^+-rich environment. **Figure 4F** shows the results of adding Mg^{2+} ions where the PONb molecules in the simulation box aggregated together. Almost all Mg^{2+} ions were adsorbed onto PONb clusters, and it was difficult to find free Mg^{2+} ions in aqueous solution.

Based on distance-based neighboring criterion(*74*), the PONbs could be divided into separate groups as clusters to analyze the aggregate state. Particles are considered connected when they fall within a specified cutoff range. Due to the stable skeleton of PONb molecular, the Nb and O atoms in PONb were considered during analysis process. Since the metal ions effect will change the molecule distance, the neighbor cutoff distance was treated as 2.30 Å to represent the PONb's aggregate state. As shown in **Figure 4B**, when the only added ion was Li^+, this possibility remained low during the whole simulation period. In the case of Mg^{2+} ions, despite the concentration of

Mg^{2+} and other ion effects, the PONbs would cluster together in a short period of time when they met with the Mg^{2+} ions, which indicated the interaction was strong and active. In short, it can be concluded that PONb molecules exhibit a strong preferred attraction to Mg^{2+} ions, and the Mg^{2+} ions would lead the cluster of PONbs.

Molecular Dynamics Simulations Video Summaries

These four videos were generated from the molecular dynamics simulation of the interaction between metal ions with PONb molecules in water. The initial snapshots were from the equilibrium state of PONb dissolved in water. Then different combinations of metal ions were randomly added into solution, in the meantime, sufficient chloride anions were also added to ensure the charge balance. The videos describe the behaviors of ions and molecules for about 50 ns. In order to clearly visualize the process, the water molecules, K ions and Cl ions in the simulation cells were not displayed in the video.

Video 3: Interaction between Pure Li and PONb

PONbs were uniformly distributed without any clustering, suggesting that the Li ions do not facilitate PONb aggregation.

Video 4: Interaction among Li, Mg (Ratio 10:1), and PONb

The analysis shows that even at a high Li: Mg ratio (10:1), the Mg ions are preferentially attracted to PONbs, promoting clustering, while the Li ions do not exhibit any significant interaction with PONbs. This demonstrates the dominant influence of Mg ions even in lower Mg concentrations.

Video 5: Interaction among Li, Mg (Ratio 1:10), and PONb

Parallel those of Video C, with Mg ions facilitating PONb clustering irrespective of their ratio to Li. The results reiterate Mg's dominant role in PONb aggregation, with the Li ions remaining non-interactive.

Video 6: Interaction between Pure Mg and PONb

PONbs immediately attract magnesium ions, leading to rapid clustering, which underscores Mg ions' pivotal role in inducing PONb aggregation.

A consistent pattern emerges showing the Mg ions are responsible for the PONb clustering whereas the Li ions do not exhibit such behavior. This trend is observed across different concentrations with varied Li-Mg ratios.

Binding Energy Calculations

Binding energy calculations were performed using the Vienna Ab initio Simulation Package (VASP)(*75-78*). Density functional theory (DFT) with the generalized gradient approximation (GGA)(*79*) was employed to calculate the electronic structure and total energies of the systems. The projector augmented-wave (PAW)(*78*) method was used to describe the core-electron interactions. During the computation process, the cut-off energy for the employed plane-wave was adjusted to 520 eV. Brillouin zone integrations were carried out using only the Γ-point(*80*). The supercell used has dimensions of (31 Å × 31 Å × 31 Å) to eliminate the periodic interaction. The water environment was defined using the VASPsol(*81, 82*) package, which incorporates solvation effects. Considering the complex interactions in this solution, geometrical optimization was conducted until all the forces acting on each atom were less than 0.05eV $Å^{-1}$.

The binding energy of the PONb in the water solution was determined by calculating the total energy of the isolated PONb, the isolated metal cations, and the combined system. The structure of PONb was converted from the experimental observations, while the full optimization in aqueous solvent was performed. The metal cations were placed at corresponding positions to connect to bridge or terminal oxygen atoms of PONb to form the PONb-cations system (as **FigureS29** shown). The binding energy was then obtained using the following equation:

$$E_{binding} = E_{POM-M} - (E_{POM} + E_M) \quad \text{(S5)}$$

where E_{POM-M} is the total energy of the PONb-cations system, E_{POM} is the total energy of the isolated PONb, and E_M is the total energy of the metal cations in aqueous solution.

DFT calculations corroborate this preference, aligning with the binding energy hierarchy. Among these four metal ions, the Mg^{2+} ions exhibit the strongest bonding strength with the oxygen atoms on PONb molecules, regardless of whether the oxygen is in a terminal or bridge position. The binding energy of K^+ ions to bridge oxygen is lower than that to terminal oxygen, consistent with experimental observations on much higher ratio of bridge site bonding. In the case of Na^+ ions, the binding energies to bridge and terminal oxygen are similar, indicating no significant preference for either connection site.

Synthesis

Synthesis of Native-PONb, $K_8[Nb_6O_{19}]{\bullet}9H_2O$. A mixture consisting of Nb_2O_5 (0.2 g, 0.75 mmol) and KOH pellets (2.24 g, 40 mmol) was combined with 20.0 ml of deionized water (DI water) in a 43 ml Teflon-lined acid digestion vessel. This reaction mixture was stirred for approximately 5 mins at room temperature to facilitate dissolution. Subsequently, the vessel was sealed in an autoclave reactor and heated to 200 °C for 72 h. Upon cooling to room temperature, the supernatant was collected, and 60 ml of methanol (MeOH) was added. The mixture was then stirred for 30 mins to precipitate out the product. The resultant white precipitate was isolated via filtration and washed three times with 10.0 ml of cold 1:1 MeOH/DI water solution. The purified white crystals were finally collected by filtration, followed by drying for 3 hours. Formula: *$K_8[Nb_6O_{19}]{\bullet}9H_2O$*, 1336.35 g/mol. Yield: 330.1 mg, 0.247 mmol, 98.8% based on *Nb*. Experimental (calculated) % mass: Nb 41.82 (41.69), K 23.37 (23.39), H_2O 12.05 (12.11).

Synthesis of the Li-PONb, $HLi_7[Nb_6O_{19}]{\bullet}13H_2O$. A 50.0 µl aliquot of 2.0 M LiCl aqueous solution was combined with 100.0 µl of a 0.01 M $K_8[Nb_6O_{19}]{\bullet}9H_2O$ aqueous solution in a 2-ml vial. The mixture was stirred for 10 seconds, resulting in a colorless solution. Over a few hours, few colorless crystals precipitated and were subsequently collected for Single crystal X-ray diffraction. The reaction was scaled up to obtain enough powder for subsequent characterization. To purify the crystals for the further characterizations, they were washed three times with 10 ml of MeOH to eliminate chloride salt impurities. Formula: *$HLi_7[Nb_6O_{19}]{\bullet}13H_2O$*, 1145.22 g/mol. Experimental (calculated) % mass: Nb 48.81 (48.73), Li 4.23 (4.25), H_2O 20.44 (20.45).

Synthesis of the Na-PONb, $H_2Na_6[Nb_6O_{19}]{\bullet}15H_2O$. A 20 µl aliquot of 2.0 M NaCl aqueous solution was combined with 100 µl of a 0.01 M $K_8[Nb_6O_{19}]{\bullet}9H_2O$ aqueous solution in a 2-ml vial. The mixture was stirred for 10 seconds, resulting in a colorless solution. Over a few minutes, colorless crystals precipitated and were subsequently collected for Single crystal X-ray diffraction. The reaction was scaled up to obtain enough powder for subsequent characterization. To purify the crystals for the further characterizations, they were washed three times with 10 ml of MeOH to eliminate chloride salt impurities. Formula: *$H_2Na_6[Nb_6O_{19}]{\bullet}15H_2O$*, 1271.61 g/mol. Experimental (calculated) % mass: Nb 43.79 (43.91), Na 10.92 (10.87), H_2O 21.33 (21.27).

Synthesis of the Mg-PONb, $Mg_4[Nb_6O_{19}]$•$16H_2O$. The synthesis of single-crystal Mg-PONb is significantly more challenging compared to Li-PONb and Na-PONb. Even a minimal addition of Mg^{2+} can instantly produce a substantial amount of low-crystallinity powder. For instance, when 20 µl of a 0.10 M $MgCl_2$ solution was added to 100 µl of a 0.01 M Native-PONb solution, a massive precipitation of powder occurred, almost completely filling the vial. To reduce the reaction rate and minimize powder formation, thereby allowing an assessment of the limited solubility of Mg^{2+} in the Native-PONb solution, lower concentrations and smaller volumes of $MgCl_2$ were used. Despite this, the addition of even 5 µl of a 0.005 M $MgCl_2$ solution still resulted in immediate turbidity. For further analysis of the product formed by adding Mg^{2+} to the Native-PONb solution, a 15 µl aliquot of 0.000625 M $MgCl_2$ aqueous solution was mixed with 100 µl of a 0.000625 M Native-PONb aqueous solution in a 2 ml vial. The mixture was stirred for 60 seconds, resulting in a clear solution. Colorless crystals precipitated after several days and were collected for SC-XRD analysis. Due to the minimal amount of Mg^{2+} added during the preparation of Mg-PONb single crystals, the structure of Mg-PONb exhibits two Mg atoms with partial occupancy. This is attributed to the input ratio of Mg^{2+}/PONb being significantly lower than the expected ratio for the Mg-PONb structure. Consequently, the chemical formula of Mg-PONb is more reliably determined from the powder samples obtained at a normal scale. Powder X-ray diffraction patterns of Mg-PONb synthesized at standard dosages (amounts used for other characterizations) are presented at **Figure S5** to indicates that the Mg-PONb single crystals obtained under highly diluted conditions exhibit a consistent structure with the powder form of Mg-PONb synthesized at standard dosages. To purify the samples for the further characterizations, they were washed three times with 10 ml of MeOH to eliminate chloride salt impurities. Formula: *$Mg_4[Nb_6O_{19}]$•$16H_2O$*, 1246.89 g/mol. Experimental (calculated) % mass: Nb 44.74 (44.72), Mg 7.79 (7.80), H_2O 22.99 (23.10).

Data S1. Crystallographic Information File (CIF) for Li-PONb.
Contains atomic coordinates, unit cell parameters, and refinement details for the Li-PONb.

Data S2. Crystallographic Information File (CIF) for Mg-PONb.
Contains atomic coordinates, unit cell parameters, and refinement details for the Mg-PONb.

Data S3. Crystallographic Information File (CIF) for Na-PONb.
Contains atomic coordinates, unit cell parameters, and refinement details for the Na-PONb.

Data S4. Crystallographic Information File (CIF) for Native-PONb.
Contains atomic coordinates, unit cell parameters, and refinement details for the Native-PONb.

REFERENCES AND NOTES

1. J. Xiao, F. F. Shi, T. Glossmann, C. Burnett, Z. Liu, From laboratory innovations to materials manufacturing for lithium-based batteries. *Nat. Energy* **8**, 329–339 (2023).

2. F. Degen, M. Winter, D. Bendig, J. Tübke, Energy consumption of current and future production of lithium-ion and post lithium-ion battery cells. *Nat. Energy* **8**, 1284–1295 (2023).

3. M. Ahuis, S. Doose, D. Vogt, P. Michalowski, S. Zellmer, A. Kwade, Recycling of solid-state batteries. *Nat. Energy* **9**, 373–385 (2024).

4. T. T. T. Trang, J. H. Zhang, J. H. Kim, A. Zargaran, J. H. Hwang, B.-C. Suh, N. J. Kim, Designing a magnesium alloy with high strength and high formability. *Nat. Commun.* **9**, 2522 (2018).

5. Z. Wu, W. A. Curtin, The origins of high hardening and low ductility in magnesium. *Nature* **526**, 62–67 (2015).

6. T. Xin, Y. Zhao, R. Mahjoub, J. Jiang, A. Yadav, K. Nomoto, R. Niu, S. Tang, F. Ji, Z. Quadir, D. Miskovic, J. Daniels, W. Xu, X. Liao, L.-Q. Chen, K. Hagihara, X. Li, S. Ringer, M. Ferry, Ultrahigh specific strength in a magnesium alloy strengthened by spinodal decomposition. *Sci. Adv.* **7**, eabf3039 (2021).

7. X.-Q. Wang, M. Qin, N.-C. Moldovan, C.-W. Su, Bubble behaviors in lithium price and the contagion effect: An industry chain perspective. *Resour. Policy* **83**, 103725 (2023).

8. T. Kanagasundaram, O. Murphy, M. N. Haji, J. J. Wilson, The recovery and separation of lithium by using solvent extraction methods. *Coordin. Chem. Rev.* **509**, 215727 (2024).

9. A. Z. Haddad, L. Hackl, B. Akuzum, G. Pohlman, J. F. Magnan, R. Kostecki, How to make lithium extraction cleaner, faster and cheaper – In six steps. *Nature* **616**, 245–248 (2023).

10. M.-p. Zheng, E.-y. Xing, X.-f. Zhang, M.-m. Li, D. Che, L.-z. Bu, J.-h. Han, C.-y. Ye, Classification and mineralization of global lithium deposits and lithium extraction technologies for exogenetic lithium deposits. *China Geol.* **6**, 547–566 (2023).

11. M. L. Vera, W. R. Torres, C. I. Galli, A. Chagnes, V. Flexer, Environmental impact of direct lithium extraction from brines. *Nat. Rev. Earth. Environ.* **4**, 149–165 (2023).

12. I. Warren, Techno-Economic Analysis of Lithium Extraction from Geothermal Brines. *Golden, CO: National Renewable Energy Laboratory*, NREL/TP-5700-79178 (2021).

13. M. Yong, M. Tang, L. Sun, F. Xiong, L. Xie, G. Zeng, X. Ren, K. Wang, Y. Cheng, Z. Li, E. Li, X. Zhang, H. Wang, Sustainable lithium extraction and magnesium hydroxide co-production from salt-lake brines. *Nat. Sustain.* **7**, 1662–1671 (2024).

14. Q. Peng, R. Wang, Z. Zhao, S. Lin, Y. Liu, D. Dong, Z. Wang, Y. He, Y. Zhu, J. Jin, L. Jiang, Extreme Li-Mg selectivity via precise ion size differentiation of polyamide membrane. *Nat. Commun.* **15**, 2505 (2024).

15. S. Xu, J. Song, Q. Bi, Q. Chen, W.-M. Zhang, Z. Qian, L. Zhang, S. Xu, N. Tang, T. He, Extraction of lithium from Chinese salt-lake brines by membranes: Design and practice. *J. Memb. Sci.* **635**, 119441 (2021).

16. P. Xu, J. Hong, X. Qian, Z.Xu, H. Xia, X. Tao, Z. Xu, Q.-Q. Ni, Materials for lithium recovery from salt lake brine. *J. Mater. Sci.* **56**, 16–63 (2021).

17. C. Liu, Y. Li, D. Lin, P.-C. Hsu, B. Liu, G. Yan, T. Wu, Y. Cui, S. Chu, Lithium extraction from seawater through pulsed electrochemical intercalation. *Joule* **4**, 1459–1469 (2020).

18. T. Zhang, W. Zheng, Q. Wang, Z. Wu, Z. Wang, Designed strategies of nanofiltration technology for Mg^{2+}/Li^{+} separation from salt-lake brine: A comprehensive review. *Desalination* **546**, 116205 (2023).

19. R. Wang, R. He, T. He, M. Elimelech, S. Lin, Performance metrics for nanofiltration-based selective separation for resource extraction and recovery. *Nat. Water* **1**, 291–300 (2023).

20. Z. Li, I.-C. Chen, L. Cao, X. Liu, K.-W. Huang, Z. Lai, Lithium extraction from brine through a decoupled and membrane-free electrochemical cell design. *Science* **385**, 1438–1444 (2024).

21. Y. Song, S. Fang, N. Xu, M. Wang, S. Chen, J. Chen, B. Mi, J. Zhu, Solar transpiration–powered lithium extraction and storage. *Science* **385**, 1444–1449 (2024).

22. B. Liu, J. Yang, X. Zhang, Q. Yang, J. Zhang, X. Li, Development and application of magnesium alloy parts for automotive OEMs: A review. *J. Magnes. Alloy* **11**, 15–47 (2023).

23. L. Chen, K. A. San, M. J. Turo, M. Gembicky, S. Fereidouni, M. Kalaj, A. M. Schimpf, Tunable metal oxide frameworks via coordination assembly of preyssler-type molecular clusters. *J. Am. Chem. Soc.* **141**, 20261–20268 (2019).

24. L. Chen, M. J. Turo, M. Gembicky, R. A. Reinicke, A. M. Schimpf, Cation-controlled assembly of polyoxotungstate-based coordination networks. *Angew. Chem. Int. Ed. Engl.* **59**, 16609–16615 (2020).

25. M. J. Turo, L. Chen, C. E. Moore, A. M. Schimpf, Co^{2+}-linked $[NaP_5W_{30}O_{110}]^{14-}$: A redox-active metal oxide framework with high electron density. *J. Am. Chem. Soc.* **141**, 4553–4557 (2019).

26. S. Zhang, W. Shi, X. Wang, Locking volatile organic molecules by subnanometer inorganic nanowire-based organogels. *Science* **377**, 100–104 (2022).

27. D.-L. Long, R. Tsunashima, L. Cronin, Polyoxometalates: Building blocks for functional nanoscale systems. *Angew. Chem. Int. Ed. Engl.* **49**, 1736–1758 (2010).

28. A. Misra, K. Kozma, C. Streb, M. Nyman, Beyond charge balance: Counter-cations in polyoxometalate chemistry. *Angew. Chem. Int. Ed. Engl.* **59**, 596–612 (2020).

29. T. Rahman, E. Petrus, M. Segado, N. P. Martin, L. N. Palys, M. A. Rambaran, C. A. Ohlin, C. Bo, M. Nyman, Predicting the solubility of inorganic ions pairs in water. *Angew. Chem. Int. Ed. Engl.* **61**, e202117839 (2022).

30. H. Zhang, A. Li, K. Li, Z. Wang, X. Xu, Y. Wang, M. V. Sheridan, H.-S. Hu, C. Xu, E. V. Alekseev, Z. Zhang, P. Yan, K. Cao, Z. Chai, T. E. Albrecht-Schönzart, S. Wang, Ultrafiltration separation of Am(VI)-polyoxometalate from lanthanides. *Nature* **616**, 482–487 (2023).

31. S. Kikkawa, Y. Fujiki, V. Chudatemiya, H. Nagakari, K. Shibusawa, J. Hirayama, N. Nakatani, S. Yamazoe, Water-tolerant superbase polyoxometalate $[H_2(Nb_6O_{19})]^{6-}$ for homogeneous catalysis. *Angew. Chem. Int. Ed. Engl.* **63**, e202401526 (2024).

32. M. Nyman, T. M. Alam, F. Bonhomme, M. A. Rodriguez, C. S. Frazer, M. E. Welk, Solid-state structures and solution behavior of alkali salts of the $[Nb_6O_{19}]^{8-}$ Lindqvist ion. *J. Clust. Sci.* **17**, 197–219 (2006).

33. Y. Hou, D. B. Fast, R. E. Ruther, J. M. Amador, L. B. Fullmer, S. R. Decker, L. N. Zakharov, M. R. Dolgos, M. Nyman, The atomic level journey from aqueous polyoxometalate to metal oxide. *J. Solid State Chem.* **221**, 418–425 (2015).

34. T. M. Anderson, S. G. Thoma, F. Bonhomme, M. A. Rodriguez, H. Park, J. B. Parise, T. M. Alam, J. P. Larentzos, M. Nyman, Lithium polyniobates. A lindqvist-supported lithium-water adamantane cluster and conversion of hexaniobate to a discrete keggin complex. *Cryst. Growth Des.* **7**, 719–723 (2007).

35. T. M. Anderson, M. A. Rodriguez, F. Bonhomme, J. N. Bixler, T. M. Alama, M. Nyman, An aqueous route to $[Ta_6O_{19}]^{8-}$ and solid-state studies of isostructural niobium and tantalum oxide complexes. *Dalton Trans.* 4517–4522 (2007).

36. I. Lindqvist, The structure of the hexaniobate Ion in $7Na_2O\cdot 6Nb_2O_5\cdot 32H_2O$. *Ark. Kemi.* **5**, 247–250 (1953).

37. E. Balogh, T. M. Anderson, J. R. Rustad, M. Nyman, W. H. Casey, Rates of oxygen-isotope exchancie between sites in the $[H_xTa_6O_{19}]^{(8-x)-}$(aq) lindqvist ion and aqueous solutions: Comparisons to $[H_xNb_6O_{19}]^{(8-x)-}$(aq). *Inorg. Chem.* **46**, 7032–7039 (2007).

38. C. A. S. Cabezas, K. Miller, S. Heo, A. Dolocan, G. LeBlanc, D. J. Milliron, Direct electrochemical deposition of transparent metal oxide thin films from polyoxometalates. *Chem. Mater.* **32**, 4600–4608 (2020).

39. R. P. Bontchev, M. Nyman, Evolution of polyoxoniobate cluster anions. *Angew. Chem. Int. Ed. Engl.* **45**, 6670–6672 (2006).

40. N. P. Martin, M. Nyman, Directional bonding in decaniobate inorganic frameworks. *Angew. Chem. Int. Ed. Engl.* **60**, 954–960 (2021).

41. M. Scepanovic, M. Grujic-Brojcin, K. Vojisavljevic, S. Bernik, T. Sreckovic, Raman study of structural disorder in ZnO nanopowders. *J. Raman Spectrosc.* **41**, 914–921 (2010).

42. T. M. Alam, M. Nyman, B. R. Cherry, J. M. Segall, L. E. Lybarger, Multinuclear NMR investigations of the oxygen, water, and hydroxyl environments in sodium hexaniobate. *J. Am. Chem. Soc.* **126**, 5610–5620 (2004).

43. T. Iizuka, K. Ogasawara, K. Tanabe, Acidic and catalytic properties of niobium pentaoxide. *Bull. Chem. Soc. Jpn.* **56**, 2927–2931 (1983).

44. M. Segado, M. Nyman, C. Bo, Aggregation patterns in low- and high-charge anions define opposite solubility trends. *J. Phys. Chem. B* **123**, 10505–10513 (2019).

45. Y. Zhang, R. Xu, L. Wang, W. Sun, Separation of magnesium from lithium in salt-lake brine through struvite precipitation. *Miner. Eng.* **180**, 107468 (2022).

46. J. Ying, M. Luo, Y. Jin, J. Yu, Selective separation of lithium from high Mg/Li ratio brine using single-stage and multi-stage selective electrodialysis processes. *Desalination* **492**, 114621 (2020).

47. C. Zhang, Y. Mu, S. Zhao, W. Zhang, Y. Wang, Lithium extraction from synthetic brine with high Mg^{2+}/Li^{+} ratio using the polymer inclusion membrane. *Desalination* **496**, 114710 (2020).

48. X. Guo, S. Hu, C. Wang, H. Duan, X. Xiang, Highly efficient separation of magnesium and lithium and high utilization of magnesium from salt lake brine by a ReactionCoupled-CoupTechnology. *Ind. Eng. Chem. Res.* **57**, 6618–6626 (2018).

49. K. T. Tran, T. Van Luong, J.-W. An, D.-J. Kang, M.-J. Kim, T. Tran, Recovery of magnesium from Uyuni salar brine as high purity magnesium oxalate. *Hydrometallurgy* **138**, 93–99 (2013).

50. C. Ma, J. Zhang, Y. Liu, L. Sun, Q. Liu, Integrated separation of precipitation and three-liquid-phase extraction for boron, lithium and magnesium from salt lake brine with high magnesium-lithium ratio. *Sep. Purif. Technol.* **347**, 127541 (2024).

51. H. Wang, Y. Zhong, B. Du, Y. Zhao, M. Wang, Recovery of both magnesium and lithium from high Mg/Li ratio brines using a novel process. *Hydrometallurgy* **175**, 102–108 (2018).

52. K.-Y. Ju, D.-F. Liu, Z.-w. Zhao, L.-H. He, W.-h. Xu, Efficiently separating Li^{+} and Mg^{2+} from brine and directly preparing Li_3PO_4 by a combination of electrochemical intercalation/deintercalation and $MgNH_4PO_4$ precipitation. *Sep. Purif. Technol.* **324**, 124643 (2023).

53. X.-Y. Nie, S.-Y. Sun, X. Song, J.-G. Yu, Further investigation into lithium recovery from salt lake brines with different feed characteristics by electrodialysis. *J. Memb. Sci.* **530**, 185–191 (2017).

54. X.-Y. Nie, S.-Y. Sun, Z. Sun, X. Song, J.-G. Yu, Ion-fractionation of lithium ions from magnesium ions by electrodialysis using monovalent selective ion-exchange membranes. *Desalination* **403**, 128–135 (2017).

55. R. He, C. Dong, S. Xu, C. Liu, S. Zhao, T. He, Unprecedented Mg^{2+}/Li^{+} separation using layer-by-layer based nanofiltration hollow fiber membranes. *Desalination* **525**, 115492 (2022).

56. R. He, S. Xu, R. Wang, B. Bai, S. Lin, T. He, Polyelectrolyte-based nanofiltration membranes with exceptional performance in Mg^{2+}/Li^{+} separation in a wide range of solution conditions. *J. Memb. Sci.* **663**, 121027 (2022).

57. D. Lu, T. Ma, S. Lin, Z. Zhou, G. Li, Q. An, Z. Yao, Q. Sun, Z. Sun, L. Zhang, Constructing a selective blocked-nanolayer on nanofiltration membrane via surface-charge inversion for promoting Li^{+} plus permselectivity over Mg^{2+}. *J. Memb. Sci.* **635**, 119504 (2021).

58. Y. Xu, H. Peng, H. Luo, Q. Zhang, Z. Liu, Q. Zhao, High performance Mg^{2+}/Li^{+} separation membranes modified by a *bis*-quaternary ammonium salt. *Desalination* **526**, 115519 (2022).

59. D. Massiot, F. Fayon, M. Capron, I. King, S. Le Calvé, B. Alonso, J.-O. Durand, B. Bujoli, Z. Gan, G. Hoatson, Modelling one- and two-dimensional solid-state NMR spectra. *Magn. Reson. Chem.* **40**, 70–76 (2002).

60. G. R. Fulmer, A. J. M. Miller, N. H. Sherden, H. E. Gottlieb, A. Nudelman, B. M. Stoltz, J. E. Bercaw, K. I. Goldberg, NMR chemical shifts of trace impurities: Common laboratory solvents, organics, and gases in deuterated solvents relevant to the organometallic chemist. *Organometallics* **29**, 2176–2179 (2010).

61. D. R. Kinney, I. S. Chuang, G. E. Maciel, Water and the silica surface as studied by variable-temperature high-resolution H-1-Nmr. *J. Am. Chem. Soc.* **115**, 6786–6794 (1993).

62. G. M. Sheldrick, SHELXT – Integrated space-group and crystal-structure determination. *Acta Crystallogr. A* **71**, 3–8 (2015).

63. A. L. Spek, PLATON SQUEEZE: A tool for the calculation of the disordered solvent contribution to the calculated structure factors. *Acta Crystallogr. C* **71**, 9–18 (2015).

64. Z. Mao, M. Rashwan, E. G. Ribó, M. Nord, L. N. Zakharov, T. W. Surta, A. Uysal, M. Nyman, Carbon dioxide capture by niobium polyoxometalate fragmentation. *J. Am. Chem. Soc.* **146**, 19489–19498 (2024).

65. M. T. Pope, A. Muller, Polyoxometalate chemistry – An old field with new dimensions in several disciplines. *Angew. Chem. Int. Ed. Engl.* **30**, 34–48 (1991).

66. N. Agmon, Isoelectronic theory for cationic radii. *J. Am. Chem. Soc.* **139**, 15068–15073 (2017).

67. Y. Marcus, Ionic-radii in aqueous-solutions. *Chem. Rev.* **88**, 1475–1498 (1988).

68. A. P. Thompson, H. M. Aktulga, R. Berger, D. S. Bolintineanu, W. M. Brown, P. S. Crozier, P. J. I. Veld, A. Kohlmeyer, S. G. Moore, T. D. Nguyen, R. Shan, M. J. Stevens, J. Tranchida, C. Trott, S. J. Plimpton, LAMMPS-a flexible simulation tool for particle-based materials modeling at the atomic, meso, and continuum scales. *Comput. Phys. Commun.* **271**, 108171 (2022).

69. S. Koneshan, J. C. Rasaiah, R. M. Lynden-Bell, S. H. Lee, Solvent structure, dynamics, and ion mobility in aqueous solutions at 25°C. *J. Phys. Chem. B* **102**, 4193–4204 (1998).

70. D. J. Price, C. L. Brooks III, A modified TIP3P water potential for simulation with Ewald summation. *J. Chem. Phys.* **121**, 10096–10103 (2004).

71. D. Boda, D. Henderson, The effects of deviations from Lorentz-Berthelot rules on the properties of a simple mixture. *Mol. Phys.* **106**, 2367–2370 (2008).

72. C.-J. Lin, J.-J. Wang, Y. Jiang, S.-L. Chen, H.-F. Li, W.-H. Zhao, Q.-R. Huang, C.-R. Rong, X.-Z. Duan, Structural properties of ions and polyelectrolytes in aqueous solutions under external electric fields: The sign effect. *Chin. J. Polym. Sci.* **42**, 1341–1352 (2024).

73. T. Darden, D. York, L. Pedersen, Particle mesh Ewald – An N.Log(N) method for Ewald sums in large systems. *J. Chem. Phys.* **98**, 10089–10092 (1993).

74. A. Stukowski, Visualization and analysis of atomistic simulation data with OVITO-the Open Visualization Tool. *Modelling Simul. Mater. Sci. Eng.* **18**, 015012 (2010).

75. G. Kresse, J. Hafner, Ab initio molecular dynamics for liquid metals. *Phys. Rev. B* **47**, 558–561 (1993).

76. G. Kresse, J. Furthmuller, Efficiency of ab-initio total energy calculations for metals and semiconductors using a plane-wave basis set. *Comput. Mater. Sci.* **6**, 15–50 (1996).

77. G. Kresse, J. Furthmuller, Efficient iterative schemes for ab initio total-energy calculations using a plane-wave basis set. *Phys. Rev. B* **54**, 11169–11186 (1996).

78. G. Kresse, D. Joubert, From ultrasoft pseudopotentials to the projector augmented-wave method. *Phys. Rev. B* **59**, 1758–1775 (1999).

79. J. P. Perdew, K. Burke, M. Ernzerhof, Generalized gradient approximation made simple. *Phys. Rev. Lett.* **77**, 3865–3868 (1996).

80. H. J. Monkhorst, J. D. Pack, Special points for Brillouin-zone integrations. *Phys. Rev. B* **13**, 5188–5192 (1976).

81. K. Mathew, V. S. C. Kolluru, S. Mula, S. N. Steinmann, R. G. Hennig, Implicit self-consistent electrolyte model in plane-wave density-functional theory. *J. Chem. Phys.* **151**, 234101 (2019).

82. S. M. R. Islam, F. Khezeli, S. Ringe, C. Plaisance, An implicit electrolyte model for plane wave density functional theory exhibiting nonlinear response and a nonlocal cavity definition. *J. Chem. Phys.* **159**, 234117 (2023).
